# Child Support Receipt and Child Poverty Among Custodial-Mother Families in Chile and Colombia: A Longitudinal Analysis

**DOI:** 10.1007/s10834-025-10074-4

**Published:** 2026-02-24

**Authors:** Laura Cuesta, Sarah Reynolds

**Affiliations:** 1 School of Social Work, Rutgers, The State University of New Jersey, 390 George Street, Room 814, New Brunswick, NJ 08901 USA; 2 School of Public Health, University of California—Berkeley, 429 University Hall, 2199 Addison St, Berkeley, CA 94720 USA

**Keywords:** Child poverty, Child support, Income support programs, Longitudinal analysis, Latin American countries

## Abstract

Children living in custodial-mother families are disproportionately poor as compared to children living with both parents. Child support from the noncustodial father is associated with lower poverty rates among custodial-mother families, suggesting that policies to promote child support payments improve the economic well-being of these families. Yet, we do not know whether child support remains a protective factor against child poverty when other anti-poverty strategies are considered, and whether this association remains significant throughout childhood, especially in middle- and low-income countries. We contribute to addressing these research gaps by investigating the following questions in Chile and Colombia: (1) How do child poverty and child support receipt change over time among children in custodial-mother families? (2) To what extent does child support protect these children against *concurrent* poverty? And (3) To what extent does child support protect these children against *future* childhood poverty? We find that chronic poverty is common among children in custodial-mother families in both countries, but a higher proportion of Colombian children remain poor throughout their childhood. In both countries child support is inconsistently received, but a higher proportion of Chilean children receive child support throughout their childhood. Child support is associated with a 6–8 percentage point decline in concurrent poverty in both countries. However, child support is associated with a decline in *future* childhood poverty only in Chile. Our findings highlight the importance of cross-national research to better understand the strengths and limitations of child support policy as a strategy to address child poverty.

## Introduction

In a wide range of countries, children in custodial-mother families (i.e., families in which a child lives with their biological or adoptive mother and not biological or adoptive father) are disproportionately poor in comparison to children living with both parents (Cuesta, 2022; Hakovirta, 2011). Child support, a monetary transfer from the noncustodial father to the custodial mother to assist with the cost of raising their children, is associated with lower poverty rates among custodial-mother families in the Americas (Cuesta et al., 2018; Meyer & Hu, 1999), Australia (Skinner et al., 2017), and several European countries (Hakovirta, 2011). This finding suggests that in a wide range of countries, policies to promote child support payments may be key to improving the economic well-being of children living with custodial mothers.

However, most research on the relationship between child support receipt and child poverty is based on a method that compares poverty rates when child support is or is not included in the family’s income. We do not know whether the lower poverty rates among custodial-mother families receiving child support are explained by child support alone, and whether child support remains a protective factor against child poverty when other strategies to avoid poverty are considered (e.g., participating in income support programs). Another limitation of this literature is that it is primarily based on cross-sectional data, so we do not know whether the association between child support receipt and child poverty—if any—remains significant throughout childhood. Furthermore, we know little about the dynamics of child poverty and child support receipt among children in custodial-mother families, especially in middle- and low-income countries.

We contribute to addressing these research gaps by using the Chilean Early Childhood Longitudinal Survey (ELPI in Spanish) and the Colombian Longitudinal Survey (ELCA in Spanish) to investigate the following questions: (1) How do child poverty and child support receipt change over time among children in custodial-mother families? (2) To what extent does child support protect children in custodial-mother families against *concurrent* poverty? And (3) To what extent does child support protect children in custodial-mother families against *future* childhood poverty? To answer the first question, we estimate the prevalence of child poverty and child support receipt among young children and examine changes in these rates over a 6- to 7-year period. To answer the second question, we conduct a series of child fixed effects models to estimate the *concurrent* association of child support receipt with child poverty. Finally, to answer the third question, we conduct a series of logit models to estimate the association of child support receipt with *future* childhood poverty. We use both absolute and relative measures of poverty to examine the extent to which child support receipt contributes to guaranteeing child’s minimum needs or brings children closer to the standard of living of the average child in Chile and Colombia.

### Conceptual Framework

Child poverty is associated with a host of risk factors that hinder child development (Brooks-Gunn & Duncan, 1997; Chaudry & Wimer, 2016; Segretin et al., 2016). Thus, parents’ ability to provide for their children is key for promoting both current and future generations’ well-being. Custodial-mother families often face more challenges than two-parent families in providing for their children. Never-married mothers tend to have fewer economic resources than partnered mothers, especially compared to those who are married (Salinas, 2011; Smock, 2000). On the other hand, married mothers often experience a decline in their economic well-being following union dissolution (Bartfeld, 2000; de Vaus et al., 2015), perhaps as a result of becoming more financially dependent on their partners after their first child is born (Musick et al., 2020). Finding affordable childcare is also difficult for custodial mothers (Ahn, 2012; Bainbridge et al., 2003) and those who manage to participate in the labor market on average make lower earnings than both men and women without children (Sigle-Rushton & Waldfogel, 2007).

Custodial mothers often rely on multiple income sources to make ends meet (Edin & Lein, 1997; Waring & Meyer, 2020). Four strategies that they may pursue to protect their children against poverty include: (1) seeking child support from the noncustodial father, (2) doing paid work, (3) applying to income support programs, or (4) relying on others (e.g., new partners or parents) for economic support. However, each of these strategies presents trade-offs for custodial mothers. For example, applying for child support and/or income support programs are bureaucratic processes that may cost both time and money (Hernanz et al., 2004; Laín & Julia, 2024), with no guaranteed outcome. Doing paid work means giving up time that custodial mothers may spent in domestic activities and may also incur additional costs (Budig et al., 2016; Stanfors et al., 2019), including childcare and transportation to the workplace. Finally, relying on others for economic support may mean the custodial mother’s preferences become subject to a household decision-making process instead of individual choice (Hanum et al., 2024; Pezzin et al., 2015).

We conceptualize custodial mothers’ decisions about which income sources to pursue to protect their children against poverty within the consumer choice theory, which posits that individuals consider the costs and benefits of various alternatives and seek outcomes that maximize benefits and minimize costs given their preferences. In light of this model, custodial mothers weigh the costs and benefits of each strategy and choose the ones that provide them with the maximum benefit at the lowest cost. For example, seeking child support from the noncustodial father takes time and money from custodial mothers (e.g., legal fees, transportation costs, loss earnings) (Cuesta et al., 2023). However, the benefits of receiving child support income are crucial to the economic well-being of these families (Cuesta et al., 2018; Salinas, 2011) so some custodial mothers may include child support as one of their strategies if they are able to maximize noncustodial father’s payment while minimizing the costs of seeking these payments.

Because children’s experiences with poverty vary throughout their childhood (Corcoran & Chaudry, 1995; Hulme & Shepherd, 2003; Narayan, 2012), with some children living in poverty temporarily and other children experiencing poverty for extended periods of time (Corcoran & Chaudry, 1995; Vakis et al., 2015), custodial mother’s choices around income sources may have implications for children well beyond protecting them against concurrent poverty (Zagel & Van Lancker, 2022). The processes by which income sources may protect children against future childhood poverty can be conceptualized within the poverty traps theoretical framework, which posits that families who are able to steadily accumulate assets will grow their way out of poverty (Barrett et al., 2016; Carter & Barrett, 2006). Drawing from this framework, child support income may enhance custodial mothers’ ability to accumulate assets, learn new skills, and strengthen their social networks, which individually or combined may increase their long-term earnings and ultimately improve the overall economic wellbeing of her children. Evidence of small transfers resulting in an exit from poverty has been found in the cash transfer literature (Daidone et al., 2015; Morton, 2019; Sabates-Wheeler et al., 2018).

Yet, consumer choice theory does not explain why different income sources may have different effects on child poverty. We frame the distinct role of child support in reducing child poverty within the mental accounting framework (Thaler, 1999), which states that individuals and households assign activities to specific accounts (Thaler, 1999). Hence, child support may play a distinct role in addressing child poverty because it is meant to be specifically assigned to child-related expenses. Empirical evidence of this process, also known as *labeling*, has been found in a number of studies looking at the effects of child support on child-related expenses (Del Boca & Flinn, 1994, 1995) and developmental outcomes (Knox, 1996), and the effects of government transfers labeled as a child benefit on child-related expenses (Kooreman, 2000), family savings (Hener, 2017), and food security and nutritional outcomes (Waidler & Devereux, 2019).

### The Chilean and Colombian Context

The rise in cohabitation, nonmarital births, and union instability (Esteve et al., 2012a, 2012b; García & Rojas, 2002; Institute for Family Studies, 2019; Salinas, 2016) has significantly changed the living arrangements of children in Chile and Colombia. About 21% of Chilean children and 37% of Colombian children live with only one parent (Institute for Family Studies, 2019) and more than 85% of these parents are women (Cuesta et al., 2019). Repartnering is relatively more common in Colombia than in Chile: less than 5% of young custodial mothers in Chile had repartnered between 2010 and 2012 (Cuesta & Reynolds, 2022) while 23% of Colombian women aged 15–39 had had two or more unions in 2015 (Profamilia, 2016). Multiple-partner fertility is also frequent among Colombian parents: in 2015, 36% of mothers aged 13–49 and 35% of fathers aged 13–59 have had children with more than one partner (Cuesta & Mogollon, 2025). In both countries, children in custodial-mother families are more likely to live in poverty than children living with both parents and the overall population of children (Cuesta et al., 2018; Herrera et al., 2011; OECD, 2021). While both countries made significant progress in poverty reduction during the first two decades of the twenty-first century, national poverty rates were substantially lower in Chile (10.8%) than in Colombia (42.5%) in 2020 (World Bank, 2022). It is important to note that national poverty rates halved over the period of both longitudinal surveys used in this study.

### Child Support Systems

In Chile, the judicial system determines and enforces child support obligations. An obligation is enforceable only if there is a court order. Parents can make child support arrangements themselves, but they are not legally binding. Children are entitled to child support from their noncustodial parent until they reach 28 years old or graduate from an undergraduate program (whichever comes first). There are some guidelines to determine the amount of the child support order: (1) the income of both parents; (2) the minimum amount for one child should be set at 40% of the country’s monthly minimum wage (MMW), which is about US $300 as of this writing; (3) the minimum amount for two or more children should be set at 30% of the MMW per child; and (4) all child support orders cannot exceed 50% of the payor’s income. Payments are typically made through a monetary transfer, though in-kind payments (such as healthcare insurance provision) are also allowed. The monetary transfer is made through a special bank account that has no handling fees and does not earn interest. Enforcement only occurs when initiated by the custodial parent, and tools include withholding from wages, jail time, international travel restrictions, driver’s license suspension, and withholding of tax refunds. A national registry of child support debtors, which was established in 2021, will allow financial institutions to intercept loans and wages from debtors in the near future.

Colombia has a hybrid system in which the determination and enforcement of child support obligations involves the judicial system and an array of public and private agencies: the National Institute of Family Well-Being, a government agency with headquarters in Bogota and 213 local branches across the country; Family Commissioners, a group of local government authorities with presence in each of the country’s 1103 towns; and conciliation centres, a set of public and private agencies that assist separated parents with extrajudicial conciliation services. Unlike Chile, child support arrangements made by the parents themselves are legally binding if they meet the following requirements: (1) all aspects are presented in writing (i.e., amount, frequency, and method of payment), (2) all aspects of the agreement are clear, and (3) they are enforceable (i.e., paternity has been established). Children are entitled to receive child support from their noncustodial parent until they turn 18 but payments can be extended until age 25 if the child is enrolled in school and financially dependent on their parents. Child support agency staff and judges are accorded a great amount of discretion in the determination of the child support obligation; they only have a few guidelines: (1) the income of both parents should be considered in the determination of the obligation, and (2) the order cannot exceed 50% of the payor’s income. Noncustodial parents are allowed to pay child support with a monetary transfer, in-kind benefits (such as healthcare insurance provision), or a combination of both. The parents determine how payments are made. Enforcement procedures are similar to those used in Chile, including a debtor registry that was created in 2021 but is not yet operating.

### Other Income Sources

Approximately half of Chilean (49.2%) and Colombian (53.1%) women aged 15 or over were doing paid work in 2019 (Economic Commission for Latin America and the Caribbean [ECLAC] & International Labor Organization [ILO], 2019). In both countries, married women are less likely to work for pay than women in cohabiting relationships and unpartnered women (Economic Commission for Latin America and the Caribbean [ECLAC] & International Labor Organization [ILO], 2019; Instituto Nacional de Estadisticas [INE], 2017; Ramm & Salinas, 2019). In both countries, women with high school degrees and at least some college education are more likely to be employed than women who do not finish high school (Economic Commission for Latin America and the Caribbean [ECLAC] & International Labor Organization [ILO], 2019). Prior estimates for Colombia indicate that on average, custodial mothers have a higher employment rate (58%) than all women (Cuesta & Meyer, 2012).

Both Chile and Colombia have income support programs directed to families with children who are living in poverty. Chile’s program, *Subsidio Unico Familiar* (Universal Family Subsidy, or SUF), is targeted to the poorest 40% of the population who have children, are pregnant, or have a disability. SUF is a monthly transfer equivalent to around US $14. Colombia’s program, *Familias en Accion* (Families in Action, or FA), is also meant for the poorest families of the country. However, unlike SUF, FA is a conditional cash transfer program. Participants must take children under age 7 to medical check-ups to receive a nutrition subsidy and must enroll children aged 6–18 in school to receive an education subsidy. The nutrition subsidy does not vary by the number of children in the family, but the average amount varies regionally, ranging from US $25 to US $30 per month. Families also can claim education subsidies for up to three children attending school, and that subsidy ranges from US $4 to US $24 per month per child (Economic Commission for Latin America and the Caribbean [ECLAC], 2020).

Finally, custodial mothers may also receive income from relatives. At least 70% of young single mothers in Chile and Colombia live in extended family households (Esteve et al., 2012a, 2012b), which suggests that mothers’ family network may play a key role in the strategies that they use to avoid child poverty. Cohabitants, such as grandparents or new romantic partners, may provide additional income, housing, and/or childcare, all of which can improve the economic well-being of mothers and their children. The high prevalence of repartnering in Colombia, and the fact that single mothers who have repartnered are less likely to have a child support arrangement (Cuesta et al., 2023), suggests there may be some trade-offs between child support from nonresident fathers and economic support from new partners.

### Prior Research and Current Study

There is no research on the dynamics of child poverty among custodial-mother families in Latin America. However, empirical evidence on changes in poverty rates in some Latin American countries (Vakis et al., 2015), including Chile (Neilson et al., 2008; Scott, 2000) and Colombia (Camacho & Muvdi, 2017), suggests that chronic poverty is a common experience in Latin America: about one in five Latin Americans were persistently poor between 2004 and 2012 (Vakis et al., 2015). Chile is included in the group of countries with the lowest rates of chronic poverty (around 10%) and Colombia is included in the group of countries with the highest rates (between 30 and 50%) (Vakis et al., 2015).

Single-country studies are consistent with these findings and add some insight on strategies that are associated with mobility from poverty. One study found that transitory poverty was more prevalent than chronic poverty in Chile (Neilson et al., 2008) and that labor income was more important to prevent poverty than other socioeconomic factors (Neilson et al., 2008). However, these processes may be different in rural areas, since a panel of 200 small farm households found that poverty reduction among these households was primarily associated with public transfers such as child allowances (Scott, 2000).

We only know of one study that has examined the dynamics of poverty in Colombia using longitudinal data: the Colombian Longitudinal Survey (whose data we use in our study). The authors found that of approximately 7000 households, about two-thirds remained in the same wealth tercile over a six-year period (Camacho & Muvdi, 2017). This suggests that chronic poverty may be more common in Colombia than in Chile, perhaps due to the larger proportion of the population in poverty. Yet, these authors also found that a higher proportion of households experienced an improvement in their wealth tercile over this six-year period, rather than a decline (Camacho & Muvdi, 2017), a hopeful finding for economic mobility.

We do not know of any study in Chile or Colombia that has examined changes in child support receipt using longitudinal data. Nevertheless, extant research conducted with cross-sectional data finds that most custodial-mother families do not receive child support in Colombia and Chile (Cuesta & Guarin, 2024; Cuesta & Meyer, 2014; Cuesta et al., 2019). The most recent estimates for Colombia show that only 1 in 4 custodial mothers received child support from a noncustodial father in 2016 (Cuesta & Guarin, 2024). Child support receipt rate among single mothers in Chile is the highest among Latin American countries for which there is microdata available (51.3%) but still nearly half of all single mothers do not receive economic support from their children’s noncustodial father (Cuesta, 2022). In Colombia, custodial mothers living in rural areas and custodial mothers with new romantic partners are less likely to have a child support arrangement (Cuesta et al., 2023); in both countries custodial mothers with high school education or less are less likely to receive any child support than those with at least some higher education (Cuesta & Meyer, 2012; Cuesta et al., 2019).

A number of studies have examined the antipoverty effectiveness of child support in Colombia using cross-sectional data (Cuesta & Meyer, 2014, 2018; Cuesta et al., 2018). These studies find that child support is associated with lower poverty rates and lower poverty gaps among those who remain poor after receiving child support. In 2008, approximately one-third of poor families who received child support were brought out of poverty by child support alone (Cuesta & Meyer, 2014). Among those who remained poor, child support reduced the poverty gap by about one-third (Cuesta & Meyer, 2014). We do not know of any study that has examined the dynamic relationship between child support receipt and child poverty in Chile or Colombia. For neither country do we have information about whether child support receipt is a protective factor against future poverty.

We build on this literature to answer the questions of our study: (1) How do child poverty and child support receipt change over time among children in custodial-mother families? We expect that the prevalence of chronic poverty will be higher among Colombian children living in custodial-mother families because chronic poverty among the general population is higher in Colombia than in Chile (Vakis et al., 2015) and custodial-mother families in Colombia are disproportionally poor with respect to the general population (Cuesta et al., 2018). Based on published statistics, we expect that child support receipt will be higher in Chile than in Colombia for any point in time, but we have no a priori hypotheses about how child support receipt changes throughout the period of early childhood in either Chile or Colombia. (2) To what extent does child support protect children in custodial-mother families against *concurrent* poverty? We hypothesize that in both countries, child support receipt will be concurrently associated with lower probability of experiencing poverty after other strategies to overcome poverty are considered. And (3) To what extent does child support protect children in custodial-mother families against *future* childhood poverty? We hypothesize that in both countries, child support receipt will be associated with lower probability of experiencing future poverty.

Our study contributes to global evidence on policy approaches to poverty among children in custodial-mother families by addressing four gaps in the literature. First, we document the transitions into and out of poverty among children in custodial-mother families. This is an important contribution because child poverty is most detrimental when it is experienced for extended periods of time (Brooks-Gunn & Duncan, 1997; Corcoran & Chaudry, 1995) and policies for addressing chronic poverty may be different from those designed to tackle transitory poverty (Hulme & Shepherd, 2003). Second, we document the extent to which children in custodial-mother families receive child support throughout their childhood, a key piece of evidence to determine whether child support is a reliable source of income that can be effective at reducing child poverty. Third, we take advantage of two longitudinal surveys to examine the extent to which the association between child support receipt and child poverty remains significant when other strategies to avoid poverty are considered. The lower poverty rates observed among custodial-mother families receiving child support (Cuesta et al., 2018; Hakovirta, 2011; Meyer & Hu, 1999; Skinner et al., 2017) may be the result of custodial mothers’ access to other income sources such as their own earnings, government income, or family support. Identifying the contribution of child support receipt to reducing child poverty will provide insight as to whether current child support policies need to be revised or complemented with other interventions. Finally, we examine the cases of Chile and Colombia, two countries with longitudinal studies and some key similarities (e.g., a high proportion of custodial-parent families) and differences (e.g., level of economic development and child support systems) that make them notable for comparison. This approach improves upon single-country studies, in which policy insights are limited to one particular context and the extent to which child support receipt may reduce child poverty across countries is less clear.

## Data and Methods

### Data

Data come from two sources: Chile’s Longitudinal Survey of Early Childhood (ELPI in Spanish) and Colombia’s Longitudinal Survey (ELCA in Spanish). The Chilean survey is a panel survey of child development, with a nationally representative sample of 15,175 children ages 0–5 in 2010. Children were selected from separate households; the survey does not have cases of siblings. Of the children interviewed in 2010, 85% and 67% were followed up in 2012 and 2017, respectively. A refresher sample of 3135 children ages 0–3 from new households was added in 2012 and 68% were followed up in 2017.

The Colombian survey is a household panel, with a nationally representative urban sample (excluding the wealthiest 3%) and a rural sample representative of small farmers. Information was collected about all household members, including children. Although there was a broader age range of children than in the Chilean survey, we restricted ages such that our Colombian sample had a parallel age structure to the Chilean sample. In 2010, 5021 children ages 0–5 from 3,970 mothers in 3754 households were in the survey. Of the children in the 2010 wave, 85% and 78% were followed up in the 2013 and 2016 waves respectively. An additional 1813 children born between the 2010 and 2013 survey rounds were present in the 2013 survey and 69% were followed up in 2016.

### Analytic Samples

We created our analytic samples of children in custodial-mother families by first determining that the biological mother—but not the biological father—lived in the child’s household. We confirmed that the mother’s marital status was separated or divorced from the biological father (though she could have been repartnered). We excluded cases in which the cause of the father’s absence made the family ineligible for child support payments; for example, in Chile, 3.7% of cases were due to migration, 1.7% due to death, 1.0% due to incarceration, and 10.2% for “other reasons.” These observations were excluded from the analysis. In Colombia, not as much data was available on cause of father absence, but we were able to exclude cases due to father death (5%) from our analytic sample. Previous research indicates the main contributor to father absence in Colombia is union dissolution (DeWaard et al., 2018).

For each child, we needed information from at least two data points to answer the study questions. Of all the children interviewed, 16,074 Chilean children and 5713 Colombian children appeared in at least two survey waves. Of these, 34% (N = 5535) of Chilean children and 28% (N = 1611) of Colombian children lived with a custodial mother at some point. In Chile, 3474 children lived with custodial mothers in multiple waves, but we excluded 314 children who lived with custodial mothers in waves 1 and 3 but lived with both biological parents in wave 2. In Colombia, 1414 children lived with a custodial mother in multiple waves, but we excluded 42 children whose residence alternated between custodial mothers and two biological parents. Thus, our analytical sample is comprised of children who lived with custodial mothers in multiple survey waves: 3160 Chilean children^1^ and 1372 Colombian children from 1181 mothers in 1145 households. We also used a subsample of children who lived with a custodial mother in all waves as a robustness check: 1139 Chilean children and 605 Colombian children from 520 mothers in 502 households.

Although this population is not representative of the general survey population, attrition rates are very similar to those reported earlier for the general population. Of the Chilean children living with custodial mothers interviewed in 2010, 86% and 67% were followed up in 2012 and 2017, respectively, and 69% of Chilean children with custodial mothers in the 2012 refresher sample were followed up in 2017. Of the Colombian children with custodial mothers included in the 2010 wave, 80% and 70% were followed up in 2013 and 2016, respectively, and 62% of Colombian children with custodial mothers added to the survey between 2013 and 2016 were followed up in 2016.

In our analysis, we do not use survey weights because they were designed to report population statistics, not statistics on custodial mothers. If custodial mothers are not distributed across the population in the same manner that the survey adjusts for its sampling, conclusions using sample weights about overall population of custodial mothers would be incorrect. Of course, this also implies that our sample of custodial mothers is not representative of all custodial mothers in the population either. Therefore, the focus of our analytical strategy is on the internal validity of our results (establishing the relationships) rather than the external validity (generalization to the population of custodial mothers) of our findings. Despite not being representative, statistic from national samples provide a useful first glimpse into these questions.

### Measures

#### Child Support Receipt

On every Chilean survey wave, the mother reported if she received child support payments from the focal child’s biological father. The amount of child support is not reported in a consistent fashion in all years, so we only use the binary variable receipt or not. On every Colombian survey wave, the household head reported if child support was received the year prior to the survey. Although we do not know to whom in the household the payment is designated, we assumed it is associated with children of the custodial mother.

#### Child’s Poverty Status

Each survey provided some information about household income. Chile provided detailed information on each household member’s wages/earnings (as well as the monetary value of payments in kind), unearned income, and government transfers. Colombia’s income measures were less specific, with the household head estimating monthly household income in a typical month for a variety of categories (e.g., wages, profits, dividends, private transfers, and non-regular income). To the income measure for Colombia, we also added the value of the government cash transfer program *Familias en Acción*; the comparable Chilean measure was already included in the income categories. Finally, for both countries we included the reported value of child support payments in the total income. We confirmed that statistics from the income data of the full set of children surveyed aligned with those from official survey documentation (Castaño Mesa, 2017; Centro Microdatos, 2010).

We classified children’s households as poor or not using two different poverty lines: the country’s national poverty line and 50% of median household income. The current methodology for calculating the Chilean national poverty line was established in 2014 based on the cost of a basket of basic-needs goods for the average impoverished household consisting of 4.3 members. Scaling this threshold for household size based on a factor of 0.7 and comparing to total household income determines if a household is poor by the national poverty line. For data gathered prior to 2014 (when this methodology was adopted), we applied the same methodology using the value of the basic-needs basket, which was calculated in 2012 and 2010. Although these were not the official poverty lines in those years, using the same methodology across all survey waves allows for more consistency in the measurement of poverty status. The Colombian national poverty lines are distinct for rural and urban areas, and they correspond to per capita household income.

The poverty line of 50% of median household income allows for a relative measure of poverty and is widely used in cross-national research on child poverty (OECD, 2021). To determine whether a child’s household is living in poverty, the income divided by the square root of household size is compared to the poverty threshold. We calculated the national median household income first by averaging per capita income of the 5th & 6th income deciles, as provided by the Center for Distributive, Labor and Social Studies (CEDLAS) and the World Bank (The Center for Distributive, Labor, and Social Studies [CEDLAS] & the World Bank, 2022). Then we multiplied this by the average household size of the third quintile and divided by the square root of the household size of the third quintile, also provided by CEDLAS and the World Bank, though by quintile instead of by decile. The poverty rates (see Table 1) are comparable to those found in prior research (Castaño Mesa, 2017; Narea, 2014).^2^

**Table 1:** Summary statistics

	Chile				Colombia				
	All	Receives CS		No CS	All		Receives CS		No CS
	Mean	Mean		Mean	Mean		Mean		Mean
Variables	(sd)	(sd)		(sd)	(sd)		(sd)		(sd)
Poor—based on national poverty threshold^a^	0.452	0.393		0.532	0.660		0.606		0.691
	(0.006)	(0.007)		(0.009)	(0.008)		(0.014)		(0.010)
Poor—based on poverty threshold set at 50% of median income^b^	0.339	0.272		0.431	0.471		0.432		0.494
	(0.005)	(0.007)		(0.009)	(0.009)		(0.014)		(0.011)
Poverty gap^c^ if poor	4768.021	4145.989		5398.223	829.059		838.749		824.253
	(60.142)	(74.481)		(92.183)	(10.406)		(17.927)		(12.780)
Poverty gap^c^	2186.856	1672.787		2874.134	544.371		504.495		566.987
	(39.098)	(43.435)		(68.665)	(9.642)		(15.983)		(12.065)
Income percentile^d^	49.224	53.112		44.027	48.327		51.567		46.489
	(0.322)	(0.411)		(0.500)	(0.490)		(0.812)		(0.610)
Total household income	16,591.834	17,770.836		15,015.586	9712.805		10,781.644		9106.613
	(168.785)	(227.190)		(248.931)	(183.772)		(366.646)		(198.133)
Receives child support	0.578	1.000		0.000	0.362		1.000		0.000
	(0.006)	(0.000)		(0.000)	(0.008)		(0.000)		(0.000)
Mother works	0.606	0.582		0.638	0.465		0.481		0.456
	(0.006)	(0.008)		(0.009)	(0.009)		(0.014)		(0.011)
Mother's wage, average amount if works	7693.453	7765.294		7603.490	6352.336		6572.233		6220.849
	(102.202)	(130.199)		(162.502)	(87.077)		(139.758)		(111.096)
Mother's wage, average amount	4660.011	4522.704		4848.252	2955.193		3161.396		2838.244
	(75.679)	(95.658)		(122.401)	(68.110)		(115.843)		(84.038)
Family receives government income	0.429	0.406		0.460	0.511		0.534		0.497
	(0.006)	(0.007)		(0.009)	(0.009)		(0.014)		(0.011)
Government income, average amount if receives	559.531	540.308		582.822	884.091		852.964		903.037
	(14.843)	(20.635)		(21.265)	(14.375)		(21.750)		(18.945)
Government income, average amount	239.895	219.481		267.883	451.417		455.337		449.194
	(7.126)	(9.304)		(11.060)	(10.592)		(16.859)		(13.572)
Other household income	10,240.543	10,869.460		9378.332	7776.957		8360.258		7446.138
	(143.124)	(194.188)		(209.486)	(181.879)		(363.401)		(196.600)
Number of children in the household	1.723	1.717		1.732	2.097	2.148		2.068	
	(0.011)	(0.014)		(0.017)	(0.021)	(0.036)		(0.027)	
Youngest child in the household is 0-2yrs	0.358	0.374		0.336	0.360	0.356		0.362	
	(0.006)	(0.007)		(0.008)	(0.008)	(0.014)		(0.010)	
Youngest child in the household is 3-4yrs	0.314	0.329		0.293	0.256	0.252		0.258	
	(0.005)	(0.007)		(0.008)	(0.008)	(0.012)		(0.009)	
Youngest child in the household is 5yrs or more	0.328	0.297		0.371	0.384	0.392		0.380	
	(0.005)	(0.007)		(0.009)	(0.008)	(0.014)		(0.011)	
Male child in the household	0.653	0.655		0.650	0.693	0.693		0.693	
	(0.006)	(0.007)		(0.009)	(0.008)	(0.013)		(0.010)	
Mother's age at 1st birth	20.618	20.596		20.647	21.671	21.865		21.561	
	(0.048)	(0.060)		(0.079)	(0.093)	(0.150)		(0.118)	
Mother has primary or less education	0.073	0.062		0.088	0.325	0.295		0.343	
	(0.003)	(0.004)		(0.005)	(0.008)	(0.013)		(0.010)	
Mother has secondary education	0.583	0.568		0.605	0.526	0.523		0.527	
	(0.006)	(0.008)		(0.009)	(0.009)	(0.014)		(0.011)	
Mother has higher education: Incomplete	0.145	0.161		0.122	0.066	0.078		0.060	
	(0.004)	(0.006)		(0.006)	(0.004)	(0.008)		(0.005)	
Mother has higher education: Complete	0.199	0.209		0.185	0.083	0.105		0.070	
	(0.005)	(0.006)		(0.007)	(0.005)	(0.009)		(0.006)	
Mother has re-partnered	0.083	0.068		0.102	0.149	0.104		0.174	
	(0.003)	(0.004)		(0.005)	(0.006)	(0.009)		(0.008)	
Other adult in the household (besides mother & step-father)	0.686	0.671		0.707	0.730	0.717		0.738	
	(0.005)	(0.007)		(0.008)	(0.008)	(0.013)		(0.010)	
Years since biological father left	4.832	4.425		5.390	4.223	3.920		4.394	
	(0.036)	(0.044)		(0.058)	(0.053)	(0.088)		(0.066)	
Urban	0.908	0.906		0.912	0.611	0.656		0.586	
	(0.003)	(0.004)		(0.005)	(0.008)	(0.014)		(0.011)	
N (data points)	7459	4313	3146		3349		1212		2137
N (children)	3160	2372	1881		1372		818		1175

*Source*: Authors’ calculations based on ELPI (Chile) and ELCA (Colombia)

*Notes*: Sample of children living with custodial mothers in at least two survey waves. All income reported in $US/year in 2011 dollars for PPP equivalency. Urban/rural dummy not available in 2017 for Chile. ^a^ In annual US, the national poverty lines for 2010, 2013, and 2016 respectively are as follows for Urban Colombia: 2052, 2232, 2290; in annual US$, the national poverty lines for 2010, 2013, and 2016 respectively are as follows for Rural Colombia: 1224, 1344, 1368. ^b^ In annual US, the 50% of median household income (equivalized) poverty lines for 2010, 2013, and 2016 respectively are as follows for Colombia: 2460, 3054, 3240. ^c^ Poverty gap from the national poverty line; 0 if above poverty line. ^d^ Calculated using the entirety of survey participants, not just custodial mothers

For robustness, we considered the poverty gap and the percentile in the income distribution as continuous outcomes. The poverty gap is the difference between household income and the poverty line; if not poor, 0 is assigned. The percentile is calculated using the income distribution from all children surveyed of comparable ages to our analytical sample, whether or not the child has a custodial mother.

#### Additional Financial Variables

We also considered three additional strategies that a custodial mother could pursue to avoid poverty: doing paid work, applying for government cash transfers for families with children, and relying on others, such as parents or a new partner, for economic support. The Chilean survey provides earnings and employment information for each mother, while the Colombian survey only has this information if the mother was the household head or partner of the household head in the first wave; in the second and third wave this information was also added for descendants. For the cases in which information on mothers’ earnings is missing,^3^ we imputed the mothers’ earning potential by region from age and education variables. As described before, both Chile’s and Colombia’s governments offer income support programs for families with children. Chile’s *Subsidio Universal Familiar* provided about $14 a month to the poorest 40% of households. In 2011 this program added a conditional component (*Assignación Familiar*—Family Bonus) for school attendance of $16 per month.^4^ Colombia’s *Familias en Acción* has a similar payment structure based on ages and grades of the children. Using the payment schedules published for each year and region, we calculated the amount each household would receive and assigned them that amount if the household indicated they were *Familías en Acción* recipients. The average recipient family receives $60 per month. Finally, we added up all other income accruing to other adults in the household besides the custodial mother (e.g., stepfather or grandparents). For Chile, the data allowed us to sum this directly, but for Colombia we calculated this by subtracting mothers’ wages and *Familías en Acción* transfers from total income.

#### Control Variables

We controlled for a number of factors that could influence child poverty and child support receipt. We included a continuous measure of the number of children born to the mother, a dichotomous measure of whether any of these children is male, and a categorical measure of the age of the youngest child: 0–2 years, 3–4 years, and 5 + years (reference category). We also controlled for categories of mother’s education: primary or less (reference category), secondary school complete, incomplete higher education, complete higher education, and a continuous measure of mother’s age at first birth. We also included a dummy variable for urban location.

Our control variable requiring the most computing is the number of years since the father left. In Chile, this question is asked directly. If the years since the father left is unknown, we substitute with the age of the youngest child. In Colombia, this question is not asked, but the date at which child support payment started is provided. We use this date to proxy years since the father left, adding an additional year if the child support agreement was made through the legal system. For those without this information, we use the age of the youngest child. Finally, for both countries, we reconciled the time differences across survey rounds; if we found discrepancies, we assumed the earlier round was correct since in that round less time would have passed since the father left, reducing recall error.

We do not control for if the mother has repartnered and if there is another adult (non-employee, non-tenant, and non-child) in the household because these are colinear with income from these sources. However, we do present summary statistics on these variables.

### Analytic Strategy

#### The Dynamics of Child Poverty and Child Support Receipt

We calculated the percentage of children in custodial mother families transitioning into and out of poverty in each country’s survey sample. We examined transitions in the short term (2010–2012/2013) and transitions in the long term (2010–2016/2017) using both measures of poverty as described earlier. We also calculated percentages of those transitioning into and out of child support receipt in the short term and in the long term.

#### Child Support Receipt Predicts Concurrent Poverty Status

We examined the concurrent association between child support receipt (CS_i t_) and child poverty (P_i t_) using an OLS child-fixed-effects approach.^5^ We controlled for maternal wages (MW_i t_), other family members’ income sources (OI_i t_), and government transfers (GT_i t_). Additionally, we controlled for a vector of child, parental, household, and community characteristics (X_i t_). We included dummies for the wave (W) in which the observation occurred and individual child fixed effects (FE_i_). The error term was e. Because there were multiple observations per child, we clustered the standard errors by child.

1 $${\mathrm{P}}_{{\text{i t}}} = {\mathrm{CS}}_{{\text{i t}}} + {\mathrm{MW}}_{{\text{i t}}} + {\mathrm{OI}}_{{\text{i t}}} + {\mathrm{GT}}_{{\text{i t}}} + {\mathrm{X}}_{{\text{i t}}} + {\mathrm{W}} + {\mathrm{FE}}_{{\mathrm{i}}} + {\mathrm{e}}$$

We examined the components of the model sequentially. First, we examined the unadjusted correlation between poverty and child support (Model 1). Second, we added the control variables (Model 2), which are included in all subsequent models. We adjusted for each additional income mechanism separately (Models 3–5) and finally included all three in the final model (Model 6). This sequential development of the model allows us to examine the extent to which custodial mothers use of other antipoverty strategies besides child support may explain the association between child support payments and child poverty.

#### Child Support Receipt Predicts Future Poverty Status

We examined the association between child support and future childhood poverty using a series of logistic regression models. We looked at whether child support receipt (CS_i t_) for child *i* in time period *t* predicts the poverty status at a time in the future (P_i tf_), controlling for poverty status at time *t* (P_i t_). Three sets of time periods for *t* and *tf* were possible: data from waves 1 and 2, data from waves 2 and 3, and data from waves 1 and 3. We used logit regression to examine if child support receipt in the first wave of the set predicted poverty in the second wave in the set. We included dummy variables indicating which set of waves was used in the observation: W_t, tf_ for whether the observations in *t* and *tf* are from wave 1 and wave 2, wave 2 and wave 3, or wave 1 and wave 3. Similarly, because there were multiple observations for many children, we clustered the standard errors by child. As in the concurrent analyses, we examined the components of the model sequentially by adding control variables and additional economic strategies used by custodial mothers. These additional variables were from the first wave of the set, which removes the possibility of reverse causality with the poverty outcome being from the second wave of the set. The error term is e.

2 $${\mathrm{P}}_{{\text{i tf}}} = {\mathrm{CS}}_{{\text{i t}}} + {\text{ MW}}_{{\text{i t}}} + {\text{ OI}}_{{\text{i t}}} + {\text{ GT}}_{{{\text{i t}} }} + {\text{ X}}_{{\text{i t}}} + {\mathrm{W}}_{{{\mathrm{t}},{\text{ tf}}}} + {\mathrm{P}}_{{\text{i t}}} + {\mathrm{e}}$$

We repeated this analysis on two subsamples of data: short term (wave 1 and wave 2) and long term (wave 1 and wave 3). These contrasts allowed us to examine whether there are differences in the association between current child support payments and future childhood poverty over a 2- to 3-year time frame or a 6- to 7-year time frame.

#### Sensitivity Analyses

We tested the sensitivity of our analyses by considering alternative sample choices. We examined the subsample of children living with a custodial mother in all waves. For the analysis on the role of child support in predicting future poverty we do an imputation exercise. Specifically, we consider what the results may have looked like should the attritors not have left the sample: in one specification all those who are lost to follow-up are added to the sample and assigned the outcome “poor” in the future period—the period for which they are missing data—and in another specification they are assigned the outcome “not poor” in the future period. Then, using these larger samples, we repeat the analyses that include all the additional economic measures and full set of controls.

We also implemented Eqs. 1 and 2 using the continuous outcomes of the poverty gap and income percentile, which provided more variation than the dichotomous poverty status outcome; these allowed us to examine general changes that may occur throughout the distribution rather than specifically at the poverty line.

#### Subgroup Analyses

We conducted subgroup analyses to examine whether the association between child support receipt and child poverty varied by custodial mother’s characteristics empirically associated with child support receipt: repartnering, coresidence with other adults, educational attainment, and location in an urban setting (Cuesta & Meyer, 2012). Additionally, for the analysis of the association between child support receipt and future childhood poverty we also considered the poverty status at baseline to examine whether the association of child support payment with future poverty was significant for the most vulnerable.

## Results

The summary statistics of our analytic samples (Table 1) indicate high levels of child poverty in both countries, using both the national poverty line and the relative poverty threshold. Poverty rates are higher using national poverty measures (45% for Chile and 66% for Colombia) than those using relative poverty measures (33% and 47% respectively). On average, the poverty gap in Chile is about 6 times the poverty gap of Colombia. In both countries, the economic insecurity of children in custodial-mother families can also be seen in that the average household income is only about two-thirds of the GDP per capita, an individual measure. The data from both countries also indicate that children in custodial-mother families have similar poverty levels as children of similar ages in the survey wave even as time passes since their biological father left (Figs. 3 and 4).^6^ Child support receipt, however, tapers with time (both within and across survey waves) since the father left in both countries (Figs. 5 and 6).

As expected, child support receipt is higher in Chile (57%) than in Colombia (36%), but in both countries poverty rates are lower among the children who receive child support. Mothers’ wages, among those who receive wages, are about half the average household income of the sample in Chile, and approximately two-thirds the average household income in Colombia. The proportion of the sample that receives government income support is 42% in Chile and 51% in Colombia. Among families who receive government income, the average amount of the support is less than 10% of the average household income of the sample in both countries.

Other household income sources comprise the majority of support for households in the sample, although in both countries the income from other household members is smaller when the family does not receive child support. With respect to demographic characteristics, custodial mothers in both countries have on average two children, though this is lower than two in Chile and above two in Colombia. Colombian mothers had their first child at slightly older ages (21.6) than did Chilean mothers (20.6). As expected, repartnering is more frequent in the Colombian sample (15%) than in the Chilean sample (8%); Appendix Table 5 shows how these rates increase across survey waves. In both countries, around 70% of households include another adult besides the mother and her partner (if she has one).

### How do Child Poverty and Child Support Receipt Change Over Time Among Children in Custodial-Mother Families?

**Fig. 1:**
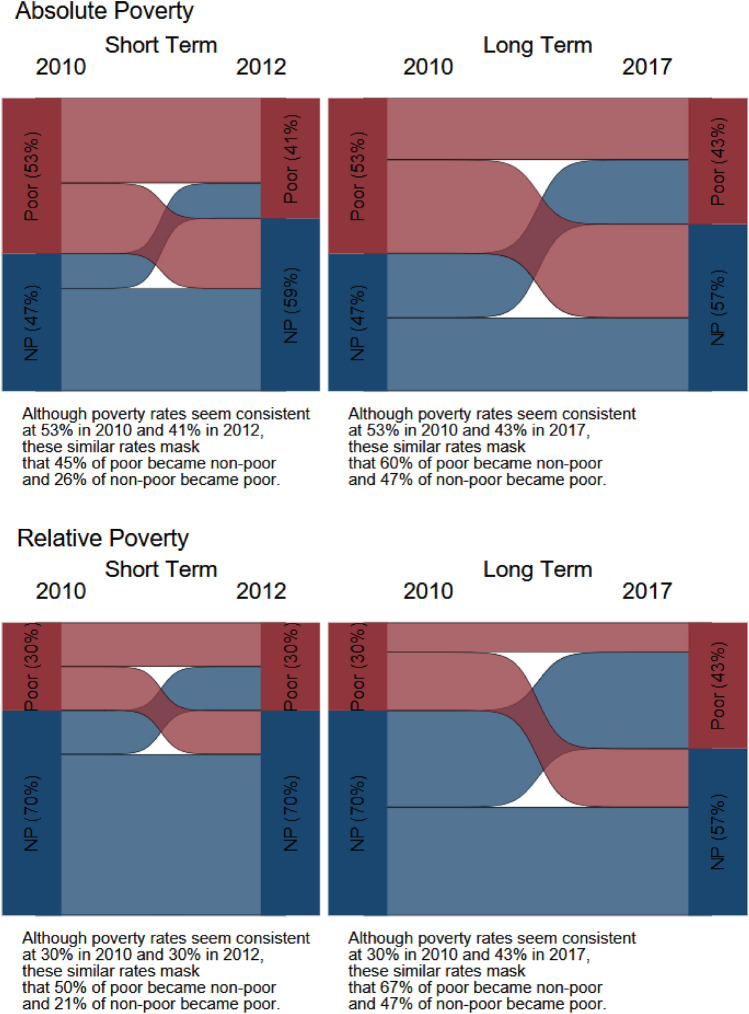

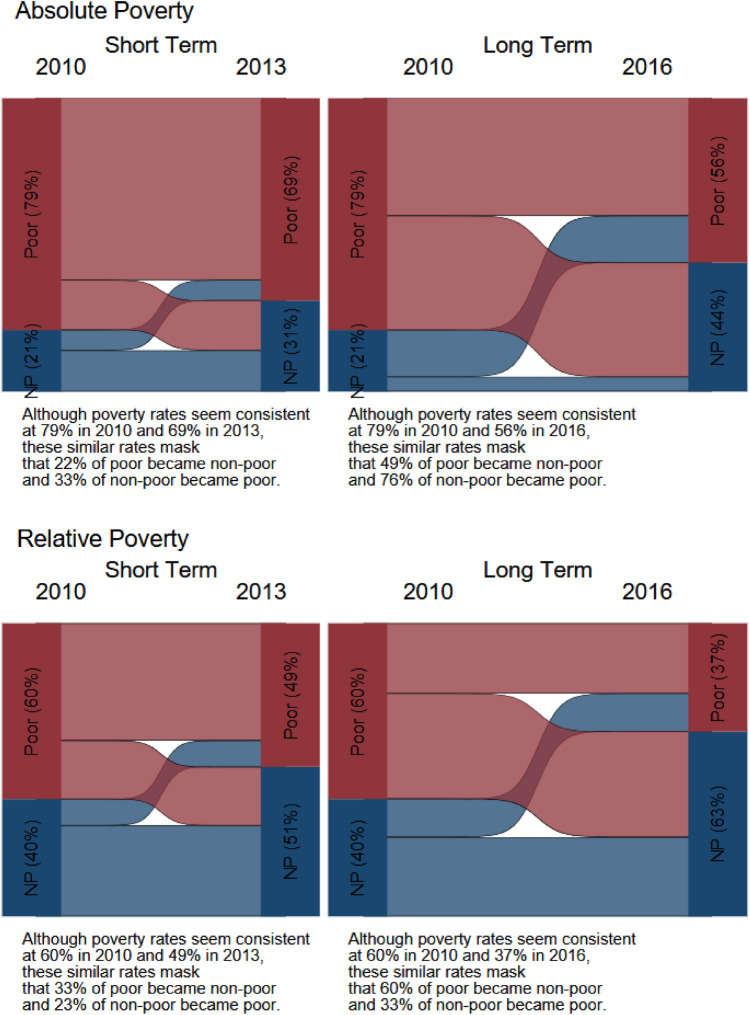
**a** Child poverty transitions among children of custodial mothers in Chile. **b** Child poverty transitions among children of custodial mothers in Colombia

We examined short- and long-term dynamics of child poverty in Fig. 1a, b, with detailed transition table in Appendix Table 6. The reduction in poverty rates suggests that the economic circumstances of children in both countries improved in the long term. However, this superficial observation hides the volatility of children entering and exiting poverty. The percentages that remained poor in the long term were near or above 50%, and as high as 62.9% (Colombia, absolute measure). In most cases, the situation for children in Colombia was worse than that in Chile: Among those who were poor in the first survey wave, a higher percentage stayed poor in Colombia than in Chile—around 15 percentage points higher using the national measures and between 4 and 15 percentage points higher using the relative measures. Among non-poor children in the first wave, in both countries 20–40% slipped into poverty in future waves. Among poor children in the first wave, about half of children in Chile exited poverty. The proportion of poor children who escaped poverty in Colombia was lower than in Chile.

**Fig. 2:**
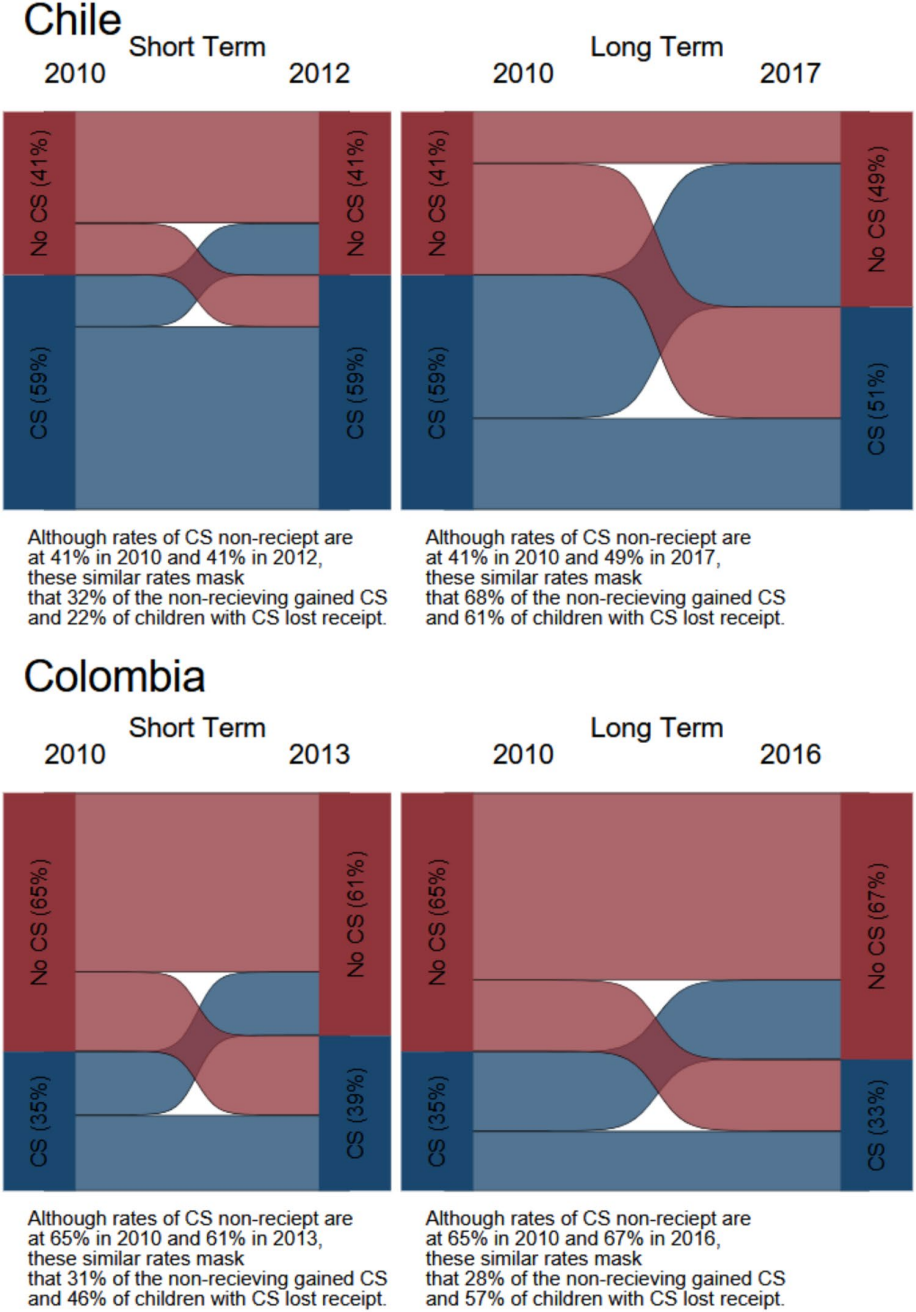
Child support receipt transitions among children of custodial mothers

**Fig. 3:**
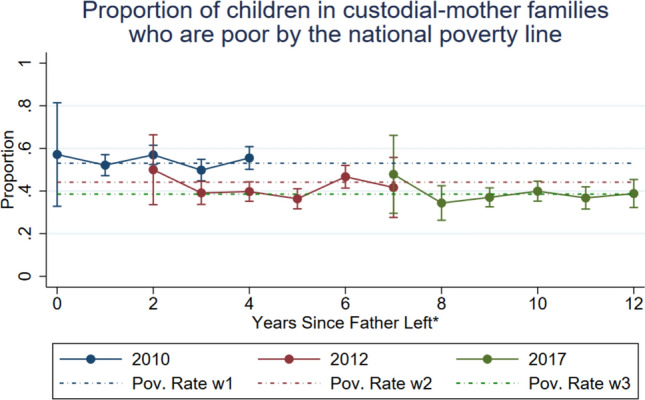
Chile

**Fig. 4:**
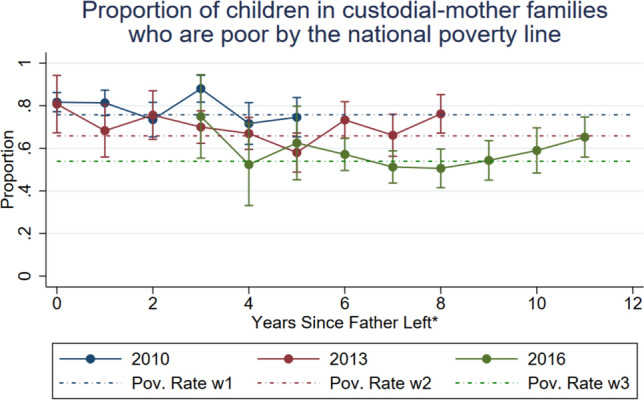
Colombia

**Fig. 5:**
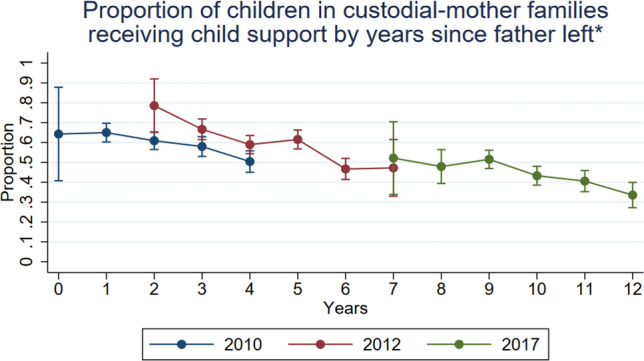
Chile

**Fig. 6:**
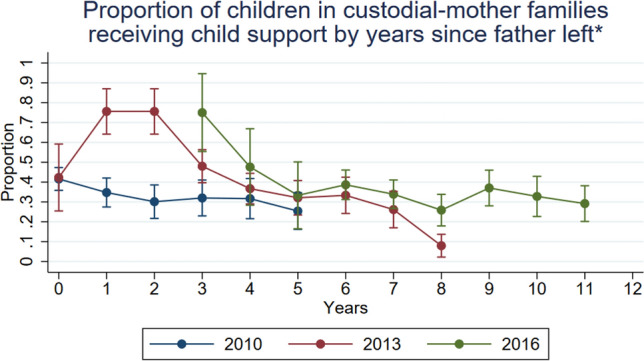
Colombia

Figure 2 shows our analyses of the dynamics of child support receipt in both the short term and the long term. (The full transition table is Appendix Table 7.) Our results suggest that in both countries, child support is not consistently provided despite similar rates of receipts across time. Among recipients of child support in the first wave (2010), the percentage of children who continued receiving child support declined in both the short term and the long term; this decline was higher in the long term, especially in Colombia, where only 39% of those who were receiving child support in 2010 continued receiving it in 2016. We also found that among those who were not receiving child support in the first wave, approximately one-third were receiving support in the short term in Chile and Colombia. However, in the long term this proportion was stable for Colombia, but lower for Chile, suggesting a falling off of payments in Chile. These high rates of transitions indicate that changes in poverty and child support payment statuses are common and allow us to estimate the models in Eqs. (1) and (2).

### To What Extent Does Child Support Protect Children in Custodial-Mother Families in Chile and Colombia Against Concurrent Poverty?

The concurrent association between child support receipt and child poverty is presented in Table 2. Our analyses using the national poverty line show that child support receipt is associated with a 6–7 percentage point decline in current poverty in both countries. These results are fairly consistent across models and remain significant after controlling for other sources of income and socioeconomic factors associated with child poverty. However, when we use the relative measure of poverty, there is no association between child support receipt and child poverty in Colombia. Results are similar when using a restricted sample of children interviewed in all survey waves (Table 8), and when we use continuous outcomes (Table 9). Yet, if there is another adult in the household (not a stepfather), concurrent child poverty is less likely than if there is no other adult in the household (Table 10). This suggests synergies between the other adult’s presence and child support payments; child support payments alone may not be sufficient in size to reduce child poverty. In addition, in both countries, child support receipt seems particularly important to prevent concurrent poverty among children whose mother has not repartnered. This finding is consistent with prior evidence indicating that custodial mothers who have a new partner are less likely to have a child support arrangement (Cuesta et al., 2023), which ultimately lowers their chances of receiving regular child support (Grall, 2020).

**Table 2:** Child support receipt predicts current poverty status

Chile	Poverty outcome: measure using national poverty threshold^a^						Poverty outcome: measure using 50% of median income^b^					
	M1	M2	M3	M4	M5	M6	M1	M2	M3	M4	M5	M6
	Coef (SE)	Coef (SE)	Coef (SE)	Coef (SE)	Coef (SE)	Coef (SE)	Coef (SE)	Coef (SE)	Coef (SE)	Coef (SE)	Coef (SE)	Coef (SE)
Child support receipt	− 0.112**	− 0.114**	− 0.125**	− 0.067**	− 0.114**	− 0.077**	− 0.132**	− 0.132**	− 0.142**	− 0.080**	− 0.132**	− 0.089**
	(0.017)	(0.017)	(0.017)	(0.017)	(0.017)	(0.016)	(0.017)	(0.017)	(0.017)	(0.016)	(0.017)	(0.016)
Log mother’s wages			− 0.015**			− 0.016**			− 0.013**			− 0.014**
			(0.001)			(0.001)			(0.001)			(0.001)
Log other household income				− 0.022**		− 0.023**				− 0.025**		− 0.026**
				(0.001)		(0.001)				(0.001)		(0.001)
Log government income					0.000	0.000					0.001	0.001
					(0.001)	(0.001)					(0.001)	(0.001)
N (observations)	7459	7459	7459	7459	7459	7459	7459	7459	7459	7459	7459	7459
N (children)	3160	3160	3160	3160	3160	3160	3160	3160	3160	3160	3160	3160
Covariates	N	Y	Y	Y	Y	Y	N	Y	Y	Y	Y	Y

Colombia	Poverty outcome: Measure using national poverty threshold^c^						Poverty outcome: Measure using 50% of median income^d^					
	M1	M2	M3	M4	M5	M6	M1	M2	M3	M4	M5	M6
	Coef (SE)	Coef (SE)	Coef (SE)	Coef (SE)	Coef (SE)	Coef (SE)	Coef (SE)	Coef (SE)	Coef (SE)	Coef (SE)	Coef (SE)	Coef (SE)
Child support receipt	− 0.068**	− 0.071**	− 0.071**	− 0.070**	− 0.068**	− 0.067**	− 0.029	− 0.028	− 0.029	− 0.027	− 0.022	− 0.022
	(0.019)	(0.019)	(0.019)	(0.019)	(0.019)	(0.019)	(0.020)	(0.020)	(0.020)	(0.020)	(0.020)	(0.020)
Log mother’s wages			− 0.001			− 0.002			− 0.001			− 0.001
			(0.001)			(0.001)			(0.001)			(0.001)
Log other household income				− 0.009**		− 0.009**				− 0.016**		− 0.015**
				(0.002)		(0.002)				(0.002)		(0.002)
Log government income					− 0.004**	− 0.004*					− 0.009**	− 0.008**
					(0.002)	(0.002)					(0.002)	(0.002)
N (observations)	3349	3349	3349	3349	3349	3349	3349	3349	3349	3349	3349	3349
N (children)	1372	1372	1372	1372	1372	1372	1372	1372	1372	1372	1372	1372
Covariates	N	Y	Y	Y	Y	Y	N	Y	Y	Y	Y	Y

*Source*: Authors’ calculations based on ELPI (Chile) and ELCA (Colombia)

*Notes*: Sample of children living with a custodial mother in at least two waves. OLS regressions with child fixed effects, standard errors clustered by child. All models include survey year dummies. Covariates are number of children, dummies for age of youngest child, male child, mother's age at first birth, mother's education dummies, years since bio-father left, and urban. ^a^ In annual US$, the national poverty lines for 2010, 2012, and 2017 respectively are as follows for Chile: 3972, 4380, 4056. ^b^ In annual US$, the 50% of median household income (equivalized) poverty lines for 2010, 2012, and 2017 respectively are as follows for Chile: 3756, 5028, 5682. ^c^ In annual US, the national poverty lines for 2010, 2013, and 2016 respectively are as follows for Rural Colombia: 1224, 1344, 1368. ^d^ In annual US$, the 50% of median household income (equivalized) poverty lines for 2010, 2013, and 2016 respectively are as follows for Colombia: 2460, 3054, 3240

### To What Extent Does Child Support Protect Children in Custodial-Mother Families in Chile and Colombia Against Future Childhood Poverty?

Table 3 shows the association between child support receipt and future childhood poverty. In Chile, child support receipt is associated with a 7-percentage point decline in the likelihood of future childhood poverty using the national poverty line, and a 6 percentage point decline in the likelihood of future childhood poverty using the relative poverty threshold. However, in Colombia the magnitude of these estimates is much lower (2 and 4 percentage point respectively) and not statistically significant. Our estimates are fairly consistent across models, even when controlling for other income sources. This suggests that child support income is not confounded with other income sources when considering its association with future childhood poverty. Mothers’ wages also are associated with a reduction in future childhood poverty in some specifications. Government transfers are positively associated with future childhood poverty, but that may be because they are only assigned to poor families. Additionally, we find that income from other household members is not associated with future childhood poverty. Results are similar to those in which we use a restricted sample of children interviewed in all survey waves (Table 11, models M1–M6). The estimated size of the reduction in poverty from child support is about half as large when attritors are included (Table 11, columns Imp. P. & Imp. N.P.) but the sign and significance remain consistent indicating our results are robust. Furthermore, the conclusions using binary outcomes align with findings using the continuous outcomes of the poverty gap and income percentile (Table 12).

**Table 3:** Child support receipt predicts future childhood poverty status

Chile	Poverty outcome at tf: Measure using national poverty threshold^a^						Poverty outcome at tf: Measure using 50% of median income^b^					
	M1	M2	M3	M4	M5	M6	M1	M2	M3	M4	M5	M6
	Coef (SE)	Coef (SE)	Coef (SE)	Coef (SE)	Coef (SE)	Coef (SE)	Coef (SE)	Coef (SE)	Coef (SE)	Coef (SE)	Coef (SE)	Coef (SE)
Child support receipt at t	− 0.067**	− 0.068**	− 0.074**	− 0.071**	− 0.065**	− 0.071**	− 0.064**	− 0.064**	− 0.071**	− 0.064**	− 0.060**	− 0.064**
	(0.016)	(0.016)	(0.017)	(0.017)	(0.016)	(0.017)	(0.016)	(0.016)	(0.016)	(0.016)	(0.016)	(0.016)
Log mother’s wages at t			− 0.004**			− 0.004**			− 0.004**			− 0.004**
			(0.001)			(0.001)			(0.001)			(0.001)
Log other household income at t				0.001		0				0		− 0.002
				(0.002)		(0.002)				(0.002)		(0.002)
Log government income at t					0.004*	0.004*					0.006**	0.006**
					(0.002)	(0.002)					(0.002)	(0.002)
N (observations)	5438	5438	5438	5438	5438	5438	5438	5438	5438	5438	5438	5438
N (children)	3160	3160	3160	3160	3160	3160	3160	3160	3160	3160	3160	3160
Covariates at t	N	Y	Y	Y	Y	Y	N	Y	Y	Y	Y	Y

Colombia	Poverty outcome at tf: Measure using national poverty threshold^c^						Poverty outcome at tf: Measure using 50% of median income^d^					
	M1	M2	M3	M4	M5	M6	M1	M2	M3	M4	M5	M6
	Coef (SE)	Coef (SE)	Coef (SE)	Coef (SE)	Coef (SE)	Coef (SE)	Coef (SE)	Coef (SE)	Coef (SE)	Coef (SE)	Coef (SE)	Coef (SE)
Child support receipt at t	− 0.005	− 0.008	− 0.008	− 0.008	− 0.024	− 0.024	− 0.038	− 0.02	− 0.022	− 0.02	− 0.038	− 0.04
	(0.024)	(0.025)	(0.025)	(0.025)	(0.025)	(0.025)	(0.024)	(0.026)	(0.026)	(0.026)	(0.026)	(0.026)
Log mother’s wages at t			0			0			− 0.004**			− 0.004**
			(0.002)			(0.002)			(0.002)			(0.002)
Log other household income at t				0.002		0.001				− 0.003		− 0.005*
				(0.002)		(0.002)				(0.002)		(0.002)
Log government income at t					0.012**	0.012**					0.011**	0.011**
					(0.002)	(0.002)					(0.002)	(0.002)
N (observations)	2582	2582	2582	2582	2582	2582	2582	2582	2582	2582	2582	2582
N (children)	1372	1372	1372	1372	1372	1372	1372	1372	1372	1372	1372	1372
Covariates at t	N	Y	Y	Y	Y	Y	N	Y	Y	Y	Y	Y

*Source*: Authors’ calculations based on ELPI (Chile) and ELCA (Colombia)

*Notes*: Sample of children living with a custodial mother in at least two waves. Logit models with standard errors clustered by child, margins at means reported. All models control for poverty status in base year, a dummy for the timeframe used, and months between surveys. Covariates from t are number of children, dummies for age of youngest child, male child, mother's age at first birth, mother's education dummies, years since bio-father left, and urban. t is wave 1 except for observations using wave 2 and wave 3, in which case t is wave 2. ^a^ In annual US$, the national poverty lines for 2010, 2012, and 2017 respectively are as follows for Chile: 3972, 4380, 4056. ^b^ In annual US$, the 50% of median household income (equivalized) poverty lines for 2010, 2012, and 2017 respectively are as follows for Chile: 3756, 5028, 5682. ^c^ In annual US, the national poverty lines for 2010, 2013, and 2016 respectively are as follows for Rural Colombia: 1224, 1344, 1368. ^d^ In annual US$, the 50% of median household income (equivalized) poverty lines for 2010, 2013, and 2016 respectively are as follows for Colombia: 2460, 3054, 3240

Dividing the sample across a variety of subgroups shows the impact of child support to be quite consistent with the main results across most categories in Chile (Table 13). One difference is that in both Chile and Colombia, future poverty reduction from child support receipt is more strongly associated among families without stepfathers. We posited that child support receipt may protect children against future poverty by enhancing custodial mothers’ ability to accumulate assets, learning new skills, or strengthening their social networks, all three processes that could improve custodial mothers’ future earnings and the overall economic wellbeing of their children. Yet, custodial mothers with new partners are less likely to have a child support arrangement (Cuesta et al., 2023), which likely affects the regularity of child support, preventing them from obtaining the long-term benefits of this transfer.

Finally, we disaggregated observations across the different time periods to see if the association between child support receipt and future childhood poverty weakened or strengthened over time (Table 4). We only found significant results of child support receipt being associated with future childhood poverty in Chile for the short term (2 years) with magnitudes of 5–7 percentage point. In the long term (7 years), magnitudes were smaller (3 percentage point) and not statistically significant. In Colombia, the magnitudes were both smaller (0–3 percentage point) and not statistically significant in both the short term (3 years) and the long term (6 years). These results did not change when we restricted our sample to children interviewed in all survey waves (Table 14).

**Table 4:** Child support receipt predicts future childhood poverty status using different timeframes for future childhood poverty

Panel A: Short term	Chile 2010–2012		Colombia 2010–2013	
	Poverty outcome		Poverty outcome	
	National Poverty Line^a^	50% Median Income^b^	National Poverty Line^c^	50% Median Income^d^
	Coef./SE	Coef./SE	Coef./SE	Coef./SE
2010 Child support receipt	− 0.074**	− 0.054**	0.001	− 0.006
	(0.024)	(0.020)	(0.037)	(0.046)
2010 Log mother’s wages	− 0.002	− 0.003*	0.005 +	− 0.002
	(0.002)	(0.001)	(0.003)	(0.003)
2010 Log other household income	− 0.002	− 0.004 +	0.001	− 0.006 +
	(0.002)	(0.002)	(0.003)	(0.004)
2010 Log government income	0.018**	0.018**	0.012**	0.013**
	(0.005)	(0.005)	(0.002)	(0.003)
N (children)	2286	2286	881	881

Panel B: Long term	Chile 2010–2017		Colombia 2010–2016	
	Poverty outcome		Poverty outcome	
	National Poverty Line^a^	50% Median Income^b^	National Poverty Line^c^	50% Median Income^d^
	Coef (SE)	Coef (SE)	Coef (SE)	Coef (SE)
2010 Child support receipt	− 0.037	− 0.033	− 0.036	− 0.011
	(0.029)	(0.030)	(0.047)	(0.048)
2010 Log mother's wages	− 0.004*	− 0.003 +	− 0.002	− 0.007*
	(0.002)	(0.002)	(0.004)	(0.003)
2010 Log other household income	0.000	0.000	0.004	0.001
	(0.003)	(0.003)	(0.004)	(0.004)
2010 Log government income	0.020**	0.019**	0.012**	0.011**
	(0.007)	(0.007)	(0.003)	(0.003)
N (children)	1308	1308	881	881

*Source*: Authors’ calculations based on ELPI (Chile) and ELCA (Colombia)

*Notes*: Sample of children living with a custodial mother in at least two waves. Logit models with standard errors clustered by child, margins at means reported. All models control for poverty status in base year, a dummy for the timeframe used, and months between surveys. Covariates from t are number of children, dummies for age of youngest child, male child, mother's age at first birth, mother's education dummies, years since bio-father left, and urban. t is wave 1 except for observations using wave 2 and wave 3, in which case t is wave 2. ^a^ In annual US$, the national poverty lines for 2010, 2012, and 2017 respectively are as follows for Chile: 3972, 4380, 4056. ^b^ In annual US$, the 50% of median household income (equivalized) poverty lines for 2010, 2012, and 2017 respectively are as follows for Chile: 3756, 5028, 5682. ^c^ In annual US, the national poverty lines for 2010, 2013, and 2016 respectively are as follows for Rural Colombia: 1224, 1344, 1368. ^d^ In annual US$, the 50% of median household income (equivalized) poverty lines for 2010, 2013, and 2016 respectively are as follows for Colombia: 2460, 3054, 3240

## Discussion

We documented transitions into and out of poverty among children in custodial-mother families in Chile and Colombia. Childhood poverty was prevalent in both countries but we found evidence supporting our hypothesis that mobility from poverty was higher in the Chilean sample than in the Colombian sample. In both countries, chronic poverty is a common experience for children in custodial-mother families, but a higher proportion of children included in the Colombian sample remain poor throughout their childhood: in Chile, about half of children who were poor in 2010 were still living in poverty in 2017. In Colombia, approximately two-thirds of children who were poor in 2010 were also poor in 2016. That the vast majority of poor children in custodial-mother families are unable to escape poverty over a 6/7-year period is concerning due to the long-lasting consequences of child poverty. To identify strategies that could inform government action, future research could use qualitative methods to examine the experiences of children in custodial-mother families who did manage to escape poverty.

Prior research using cross-sectional data has shown that a significant proportion of children in custodial-mother families in Chile and Colombia do not receive child support (Cuesta, 2022; Cuesta & Guarin, 2024; Cuesta & Meyer, 2014). We found evidence of substantial instability in child support receipt over the 6/7-year period of observation, especially in Colombia. Among the children in the Colombian sample who were receiving any support in 2010, only 39% were still receiving child support in the long term (i.e., 2016). A comparable figure for Chile in 2017 was higher (60.3%), indicating the Chilean children in the sample are more likely to receive child support consistently during their early childhood. We also found that the proportion of children who transitioned out of child support receipt in the long term was lower in Chile (20–40%) than in Colombia (40–60%). Yet, the proportion of children who became recipients of this transfer was about one-third of those who were not receiving any support in 2010 in both countries.

We found support to our hypothesis that child support was associated with a reduction in concurrent poverty in both Chile (7–8 percentage points) and Colombia (2–6 percentage points), though the reduction in Colombia using the relative measure (2 percentage points) was not significant. These findings improve upon prior research on the antipoverty effectiveness of child support relying on cross-sectional data analyses (Cuesta & Meyer, 2014; Cuesta et al., 2018). However, we only found partial support to our hypothesis that child support protects children against future childhood poverty. We had hypothesized that child support income could enhance custodial mothers’ earnings in the long term by enhancing their ability to accumulate assets, learning new skills, or strengthening their social networks. In Chile, child support receipt was associated with a reduction in future childhood poverty by 6–7 percentage points, but this was found only to be the case in the short term when considering the national poverty line (2 years) and not for the long term (7 years) nor for the relative poverty measure. In no instances did we find that child support receipt protected Colombian children against future childhood poverty. Our findings call for countries to collect detailed data on child support payments (e.g., regularity, timing, and quantity) to better understand the mechanisms behind the associations we have documented. This data will support future research in determining why child support income is not better protecting children against future poverty in these countries.

In our heterogeneity analyses, we found differences in the direction of the associations between child support receipt and concurrent poverty by custodial mother’s education (i.e., children with mothers with more than secondary education versus children with less educated mothers) in Colombia, and between child support receipt and future childhood poverty by custodial mother’s repartnering status (i.e., children with stepfathers versus children without stepfathers) in both countries. Explaining these differences is beyond the scope of the data used in this study, but future research using mode detailed information on child support could explore how this transfer is used or earmarked both in general and in these specific subsamples.

The different results for Chile and Colombia may be explained by differences in the labor market, the child support system, and family demography of these countries. In 2015, almost half of Colombians doing paid work were employed in the informal economy, while in Chile only one quarter was working in the informal sector (Fedesarrollo, 2016; ILO, 2019). That Chile has a much higher proportion of formal employment than Colombia may facilitate the establishment of child support orders and regularity of payments. Moreover, the Chilean child support system requires noncustodial parents to make payments through a special bank account that has no handling fees, which may also contribute to regularity of payments. Repartnering and multiple-partner fertility are also lower in Chile, which means child support orders may be relatively easier to establish and children may receive relatively higher amounts of support: noncustodial fathers do not have to spread income support around as many offspring in as many families. Notably, the direction of the association between child support receipt and future poverty was distinct between children living with stepfathers and children living without stepfathers in both countries. As discussed before, future research examining how child support is used or earmarked both in general and in these specific subsamples is warranted.

While Chile and Colombia have experienced a significant decline in their national poverty rates, the high incidence of chronic poverty among children in custodial-mother families calls for more ambitious interventions. Both countries have income support programs for families with children but neither of them incorporates strategies to specifically address the unique challenges faced by custodial-mother families. Providing additional services for these mothers, such as legal counseling on their child support cases, affordable childcare programs, and transportation subsidies may be a good place to start. A more ambitious policy approach could include a public guarantee of child support that custodial-mother families can receive when noncustodial fathers are unemployed, have low earnings, or are otherwise unable to provide financial support to their children. Income support programs successfully implemented during the pandemic in Latin America may provide further insight on the best approach to this issue (Menezes-Filho et al., 2021; Nazareno & de Castro Galvao, 2023). Concurrent poverty is reduced by child support payments, but future childhood poverty is not, suggesting that sustaining child support payments throughout childhood and adolescence is likely to be an important social policy that is currently underutilized.

Our findings should be interpreted in light of the following limitations. First, the surveys are not nationally representative of children of custodial mothers, but of children (Chile) and households (Colombia). Thus, our descriptive statistics are not nationally representative. However, our focus was not on demonstrating the external validity of our findings but rather providing empirical support to the hypothesized associations between child support receipt and child poverty in these two Latin American countries. Secondly, the data do not allow us to examine the amount of child support paid, which would have offered a more precise calculation of poverty reduction. We also do not have data on key characteristics of child support arrangements for Chile: whether they were court-ordered or informally arranged. There may be differences in findings around the circumstances of these different types of arrangements. Additionally, there may be other non-monetary benefits associated with child support payments that confound our findings: for example, mothers who receive child support may maintain social networks with the child's father and his family, which could allow for additional support for childcare for the mother to work or contacts for employment.

Nevertheless, this study provides new knowledge on the dynamic relationship between child support receipt and child poverty in two Latin American countries. We found that child support is a key protective factor against child poverty in both Chile and Colombia, which suggests policies to encourage child support payments are an important strategy to improve the economic well-being of children in custodial-mother families in both countries. Yet, the null relationship between child support receipt and the relative measure of poverty suggests child support receipt merely contributes to guaranteeing child’s minimum needs in Colombia. Bringing Colombian children in custodial-mother families closer to the standard of living of the average child in the country cannot be achieved by child support alone. Moreover, that there is no association between child support receipt and future poverty in Colombia—perhaps because of the substantial decline in the proportion of children receiving this transfer in the long term—suggests that Colombian policymakers need to consider alternative approaches to protect against child poverty among custodial-mother families. Finally, these differences between Chile and Colombia highlight the importance of cross-national research to better understand the strengths and limitations of child support as a strategy to address child poverty among custodial-mother families.

## Data Availability

With the exception of the *comuna* (municipality) variable, ELPI is publicly available for download: http://observatorio.ministeriodesarrollosocial.gob.cl/elpi-primera-ronda (2010) and http://observatorio.ministeriodesarrollosocial.gob.cl/elpi-segunda-ronda (2012).

## Appendix

See Figs. 3, 4, 5, 6 and Tables 5, 6, 7, 8, 9, 10, 11, 12, 13, 14.

**Table 5:** Summary statistics. Custodial mothers in multiple waves

Years that child appears in survey	Chile			Colombia		
	2010	2012	2017	2010	2012	2017
	Mean (sd)	Mean (sd)	Mean (sd)	Mean (sd)	Mean (sd)	Mean (sd)
Poor—based on national poverty threshold^a^	0.541	0.419	0.392	0.773	0.678	0.542
	(0.010)	(0.009)	(0.011)	(0.014)	(0.013)	(0.015)
Poor—based on poverty threshold set at 50% of median income^b^	0.306	0.313	0.417	0.573	0.463	0.395
	(0.009)	(0.008)	(0.011)	(0.016)	(0.014)	(0.015)
Poverty gap^c^ if poor	4577.228	4582.736	5382.866	946.300	764.667	780.854
	(88.131)	(94.171)	(144.997)	(18.277)	(15.679)	(20.142)
Poverty gap^c^	2477.856	1943.204	2189.734	731.095	514.052	420.350
	(66.296)	(57.707)	(84.136)	(19.150)	(14.468)	(15.996)
Income Percentile^d^	48.451	50.252	48.639	45.785	49.140	49.541
	(0.554)	(0.512)	(0.624)	(0.929)	(0.776)	(0.857)
Total Household Income	14,255.076	18,279.850	16,977.344	6947.528	9918.951	11,840.233
	(261.594)	(272.203)	(348.759)	(309.501)	(251.325)	(379.764)
Receives child support	0.603	0.621	0.485	0.327	0.377	0.374
	(0.010)	(0.009)	(0.011)	(0.015)	(0.013)	(0.015)
Mother works	0.541	0.602	0.690	0.694	0.328	0.433
	(0.010)	(0.009)	(0.010)	(0.015)	(0.013)	(0.015)
Mother's wage, average amount if works	5830.374	7481.301	9751.427	6250.015	6302.540	6538.080
	(143.848)	(139.943)	(230.822)	(109.808)	(193.477)	(167.676)
Mother's wage, average amount	3153.864	4507.290	6723.785	4337.152	2065.619	2833.566
	(97.430)	(107.657)	(188.263)	(120.953)	(103.394)	(121.905)
Family receives government income	0.369	0.593	0.256	0.479	0.527	0.517
	(0.010)	(0.009)	(0.010)	(0.016)	(0.014)	(0.015)
Government income, average amount if receives	0.000	573.505	1494.977	493.011	1157.740	861.185
	(0.000)	(8.130)	(70.173)	(1.011)	(24.984)	(23.954)
Government income, average amount	0.000	340.345	383.213	236.289	610.637	445.522
	(0.000)	(7.058)	(23.127)	(8.048)	(20.697)	(17.960)
Other household income	10,124.671	11,639.486	8303.249	5825.739	7780.280	9448.248
	(248.775)	(236.875)	(249.167)	(310.085)	(251.627)	(376.704)
Number of children in the household	1.571	1.697	1.947	2.000	2.098	2.179
	(0.018)	(0.017)	(0.022)	(0.041)	(0.035)	(0.036)
Youngest child in the household is 0-2yrs	0.661	0.256	0.139	0.614	0.345	0.159
	(0.010)	(0.008)	(0.008)	(0.016)	(0.013)	(0.011)
Youngest child in the household is 3-4yrs	0.339	0.449	0.081	0.284	0.280	0.203
	(0.010)	(0.009)	(0.006)	(0.015)	(0.012)	(0.012)
Youngest child in the household is 5yrs or more	0.000	0.294	0.779	0.102	0.375	0.638
	(0.000)	(0.008)	(0.009)	(0.010)	(0.013)	(0.015)
Male child in the household	0.626	0.652	0.687	0.683	0.691	0.703
	(0.010)	(0.009)	(0.010)	(0.015)	(0.013)	(0.014)
Mother's age at 1st birth	20.541	20.584	20.760	21.482	21.550	21.977
	(0.083)	(0.075)	(0.096)	(0.172)	(0.144)	(0.170)
Mother has primary or less education	0.128	0.003	0.110	0.311	0.487	0.144
	(0.007)	(0.001)	(0.007)	(0.015)	(0.014)	(0.011)
Mother has secondary education	0.624	0.572	0.551	0.637	0.287	0.716
	(0.010)	(0.009)	(0.011)	(0.016)	(0.012)	(0.014)
Mother has higher education: Incomplete	0.166	0.141	0.125	0.029	0.089	0.071
	(0.008)	(0.006)	(0.007)	(0.005)	(0.008)	(0.008)
Mother has higher education: Complete	0.082	0.285	0.215	0.023	0.137	0.068
	(0.006)	(0.008)	(0.009)	(0.005)	(0.010)	(0.008)
Mother has re-partnered	0.039	0.076	0.146	0.091	0.152	0.193
	(0.004)	(0.005)	(0.008)	(0.009)	(0.010)	(0.012)
Other adult in the household (besides mother & step-father)	0.786	0.706	0.533	0.776	0.733	0.688
	(0.008)	(0.008)	(0.011)	(0.014)	(0.012)	(0.014)
Years since biological father left	2.377	3.951	9.136	1.792	3.745	6.881
	(0.023)	(0.029)	(0.039)	(0.055)	(0.066)	(0.078)
Urban	0.910	0.909	0.905	0.572	0.612	0.644
	(0.006)	(0.005)	(0.007)	(0.016)	(0.013)	(0.014)
N	2455	2991	2013	941	1312	1096

*Source*: Authors’ calculations based on ELPI (Chile) and ELCA (Colombia)

*Notes*: All income reported in $US/year in 2011 dollars for PPP equivalency. Urban/rural dummy not available in 2017 for Chile. ^a^ In annual US$, the national poverty lines for 2010, 2012, and 2017 respectively are as follows for Chile: 3972, 4380, 4056. ^b^ In annual US$, the 50% of median household income (equivalized) poverty lines for 2010, 2012, and 2017 respectively are as follows for Chile: 3756, 5028, 5682. ^c^ In annual US, the national poverty lines for 2010, 2013, and 2016 respectively are as follows for Rural Colombia: 1224, 1344, 1368. ^d^ In annual US$, the 50% of median household income (equivalized) poverty lines for 2010, 2013, and 2016 respectively are as follows for Colombia: 2460, 3054, 3240

**Table 6:** Poverty transition tables

Chile															
Poverty measure using national poverty threshold^a^															
Short term								Long term							
N = 1139	Year 2012							N = 1139		Year 2017					
		Not poor	Row %	Poor	Row %	Total	Row %			Not poor	Row %	Poor	Row %	Total	Row %
**Year 2010**	**Not poor**	**401**	75.9	**127**	24.1	**528**	100	**Year 2010**	**Not poor**	**368**	69.7	**160**	30.3	**528**	100
	Column %	59.3		27.4		46.4			Column %	52.1		37.0		46.4	
	**Poor**	**275**	45.0	**336**	55.0	**611**	100		**Poor**	**339**	55.5	**272**	44.5	**611**	100
	Column %	40.7		72.6		53.6			Column %	47.9		63.0		53.6	
	**Total**	**676**	59.4	**463**	40.6	**1139**	100		**Total**	**707**	62.1	**432**	37.9	**1139**	100
	Column %	100		100		100			Column %	100		100		100	
Poverty threshold set at 50% of median income^b^															
**N = 1139**		**Year 2012**						**N = 1139**				**Year 2017**			
		**Not poor**	Row %	**Poor**	Row %	**Total**	Row %			**Not poor**	Row %	**Poor**	Row %	**Total**	Row %
**Year 2010**	**Not poor**	**625**	77.9	**177**	22.1	**802**	100	**Year 2010**	**Not poor**	**525**	65.5	**277**	34.5	**802**	100
	Column %	78.8		51.2		70.4			Column %	77.1		60.5		70.4	
	**Poor**	**168**	49.9	**169**	50.1	**337**	100		**Poor**	**156**	46.3	**181**	53.7	**337**	100
	Column %	21.2		48.8		29.6			Column %	22.9		39.5		29.6	
	**Total**	**793**	69.6	**346**	30.4	**1139**	100		**Total**	**681**	59.8	**458**	40.2	**1139**	100
	Column %	100		100		100			Column %	100		100		100	

Colombia															
Poverty measure using national poverty threshold^c^															
**Short Term**								**Long Term**							
**N = 605**				**Year 2013**				**N = 605**				**Year 2017**			
		**Not poor**	Row %	**Poor**	Row %	**Total**	Row %			**Not poor**	Row %	**Poor**	Row %	**Total**	Row %
**Year 2010**	**Not poor**	**83**	66.4	**42**	33.6	**125**	100	**Year 2010**	**Not poor**	**86**	68.8	**39**	31.2	**125**	100
	Column %	44.4		10.0		20.7			Column %	32.6		11.4		20.7	
	**Poor**	**104**	21.7	**376**	78.3	**480**	100		**Poor**	**178**	37.1	**302**	62.9	**480**	100
	Column %	55.6		90.0		79.3			Column %	67.4		88.6		79.3	
	**TOTAL**	**187**	30.9	**418**	69.1	**605**	100		**TOTAL**	**264**	43.6	**341**	56.4	**605**	100
	Column %	100		100		100			Column %	100		100		100	
**Poverty threshold set at 50% of median income** ^**d**^															
**N = 605**				**Year 2013**				**N = 605**			**Year 2017**				
		**Not poor**	Row %	**Poor**	Row %	**Total**	Row %			**Not poor**	Row %	**Poor**	Row %	**Total**	Row %
**Year 2010**	**Not poor**	**190**	77.9	**54**	22.1	**244**	100	**Year 2010**	**Not poor**	**187**	76.6	**57**	23.4	**244**	100
	Column %	61.7		18.2		40.3			Column %	54.7		21.7		40.3	
	**Poor**	**118**	32.7	**243**	67.3	**361**	100		**Poor**	**155**	42.9	**206**	57.1	**361**	100
	Column %	38.3		81.8		59.7			Column %	45.3		78.3		59.7	
	**TOTAL**	**308**	50.9	**297**	49.1	**605**	100		**TOTAL**	**342**	56.5	**263**	43.5	**605**	100
	Column %	100		100		100			Column %	100		100		100	

*Source*: Authors’ calculations based on ELPI (Chile) and ELCA (Colombia)

*Notes*: Sample of children living with custodial mothers in all waves. Darker quadrants indicate those transitioning into or out of poverty. Numbers in bold are N; numbers in standard type are percentages. ^a^ In annual US$, the national poverty lines for 2010, 2012, and 2017 respectively are as follows for Chile: 3972, 4380, 4056. ^b^ In annual US$, the 50% of median household income (equivalized) poverty lines for 2010, 2012, and 2017 respectively are as follows for Chile: 3756, 5028, 5682. ^c^ In annual US, the national poverty lines for 2010, 2013, and 2016 respectively are as follows for Rural Colombia: 1224, 1344, 1368. ^d^ In annual US$, the 50% of median household income (equivalized) poverty lines for 2010, 2013, and 2016 respectively are as follows for Colombia: 2460, 3054, 3240

**Table 7:** Child support receipt transition tables

Chile															
Short term								Long term							
**N = 1139**		**Year 2012**						**N = 1139**		**Year 2017**					
		**No CS**	Row %	**CS**	Row %	**Total**	Row %			**No CS**	Row %	**CS**	Row %	**Total**	Row %
**Year 2010**	**No CS**	**321**	68.7	**146**	31.3	**467**	100	**Year 2010**	**No CS**	**364**	77.9	**103**	22.1	**467**	100
	Column %	68.4		21.8		41.0			Column %	57.7		20.3		41.0	
	**CS**	**148**	22.0	**524**	78.0	**672**	100		**CS**	**267**	39.7	**405**	60.3	**672**	100
	Column %	31.6		78.2		59.0			Column %	42.3		79.7		59.0	
	**TOTAL**	**469**	41.2	**670**	58.8	**1139**	100		**TOTAL**	**631**	55.4	**508**	44.6	**1139**	100
	Column %	100		100		100			Column %	100		100		100	

Colombia															
Short term								Long term							
**N = 605**		**Year 2013**						**N = s605**		**Year 2016**					
		**No CS**	Row %	**CS**	Row %	**Total**	Row %			**No CS**	Row %	**CS**	Row %	**Total**	Row %
**Year 2010**	**No CS**	**274**	69.4	**121**	30.6	**395**	100	**Year 2010**	**No CS**	**265**	67.1	**130**	32.9	**395**	100
	Column %	73.9		51.7		65.3			Column %	67.4		61.3		65.3	
	**CS**	**97**	46.2	**113**	53.8	**210**	100		**CS**	**128**	61.0	**82**	39.0	**210**	100
	Column %	26.1		48.3		34.7			Column %	32.6		38.7		34.7	
	**Total**	**371**	61.3	**234**	38.7	**605**	100		**Total**	**393**	65.0	**212**	35.0	**605**	100
	Column %	100		100		100			Column %	100		100		100	

*Source*: Authors’ calculations based on ELPI (Chile) and ELCA (Colombia)

*Notes*: Sample of children living with custodial mothers in all waves. Darker quadrants indicate those transitioning into or out of child support. Numbers in bold are N; numbers in standard type are percentages

**Table 8:** Child support receipt predicts current poverty status. Sensitivity analysis to sample sizes

Chile	Poverty outcome: Measure using national poverty threshold^a^						Poverty outcome: Measure using 50% of median income^b^					
	M1	M2	M3	M4	M5	M6	M1	M2	M3	M4	M5	M6
	Coef (SE)	Coef (SE)	Coef (SE)	Coef (SE)	Coef (SE)	Coef (SE)	Coef (SE)	Coef (SE)	Coef (SE)	Coef (SE)	Coef (SE)	Coef (SE)
Child support receipt	− 0.123**	− 0.127**	− 0.136**	− 0.080**	− 0.127**	− 0.089**	− 0.139**	− 0.142**	− 0.150**	− 0.091**	− 0.142**	− 0.098**
	(0.024)	(0.024)	(0.023)	(0.023)	(0.024)	(0.022)	(0.024)	(0.024)	(0.023)	(0.023)	(0.024)	(0.022)
Log mother’s wages			− 0.015**			− 0.015**			− 0.013**			− 0.014**
			(0.001)			(0.001)			(0.001)			(0.001)
Log other household income				− 0.024**		− 0.025**				− 0.027**		− 0.027**
				(0.002)		(0.002)				(0.002)		(0.002)
Log government income					0	− 0.001					0.001	0
					(0.002)	(0.002)					(0.002)	(0.002)
N (observations)	3417	3417	3417	3417	3417	3417	3417	3417	3417	3417	3417	3417
N (children)	1139	1139	1139	1139	1139	1139	1139	1139	1139	1139	1139	1139
Covariates	N	Y	Y	Y	Y	Y	N	Y	Y	Y	Y	Y

Colombia	Poverty outcome: Measure using national poverty threshold^c^						Poverty outcome: Measure using 50% of median income^d^					
	M1	M2	M3	M4	M5	M6	M1	M2	M3	M4	M5	M6
	Coef (SE)	Coef (SE)	Coef (SE)	Coef (SE)	Coef (SE)	Coef (SE)	Coef (SE)	Coef (SE)	Coef (SE)	Coef (SE)	Coef (SE)	Coef (SE)
Child support receipt	− 0.070**	− 0.074**	− 0.074**	− 0.077**	− 0.072**	− 0.074**	− 0.021	− 0.025	− 0.025	− 0.028	− 0.02	− 0.024
	(0.025)	(0.025)	(0.025)	(0.025)	(0.025)	(0.025)	(0.026)	(0.026)	(0.026)	(0.025)	(0.026)	(0.025)
Log mother’s wages			− 0.001			− 0.002			− 0.001			− 0.002
			(0.001)			(0.001)			(0.002)			(0.002)
Log other household income				− 0.009**		− 0.009**				− 0.015**		− 0.015**
				(0.002)		(0.002)				(0.002)		(0.002)
Log government income					− 0.004	− 0.003					− 0.007**	− 0.006**
					(0.002)	(0.002)					(0.002)	(0.002)
N (observations)	1815	1815	1815	1815	1815	1815	1815	1815	1815	1815	1815	1815
N (children)	605	605	605	605	605	605	605	605	605	605	605	605
Covariates	N	Y	Y	Y	Y	Y	N	Y	Y	Y	Y	Y

*Source*: Authors’ calculations based on ELPI (Chile) and ELCA (Colombia)

*Notes*: Sample of children in custodial-mother families in all waves. OLS regressions with child and wave fixed effects, standard errors clustered by child. All models include survey year dummies. Covariates are number of children, dummies for age of youngest child, male child, mother's age at first birth, mother's education dummies, years since bio-father left, and urban. ^a^ In annual US$, the national poverty lines for 2010, 2012, and 2017 respectively are as follows for Chile: 3972, 4380, 4056. ^b^ In annual US$, the 50% of median household income (equivalized) poverty lines for 2010, 2012, and 2017 respectively are as follows for Chile: 3756, 5028, 5682. ^c^ In annual US, the national poverty lines for 2010, 2013, and 2016 respectively are as follows for Rural Colombia: 1224, 1344, 1368. ^d^ In annual US$, the 50% of median household income (equivalized) poverty lines for 2010, 2013, and 2016 respectively are as follows for Colombia: 2460, 3054, 3240

**Table 9:** Child support receipt predicts current continuous outcomes

Chile	Continuous outcome: Poverty gap from national poverty threshold^a^						Continuous outcome: Percentile in the income distribution^b^					
	M1	M2	M3	M4	M5	M6	M1	M2	M3	M4	M5	M6
	Coef (SE)	Coef (SE)	Coef (SE)	Coef (SE)	Coef (SE)	Coef (SE)	Coef (SE)	Coef (SE)	Coef (SE)	Coef (SE)	Coef (SE)	Coef (SE)
Child support receipt	− 910.890**	− 932.609**	− 1014.728**	− 464.948**	− 935.089**	− 534.042**	6.915**	7.024**	7.663**	3.196**	6.995**	3.679**
	(127.735)	(127.063)	(119.369)	(118.008)	(127.121)	(108.708)	(0.905)	(0.903)	(0.843)	(0.830)	(0.903)	(0.754)
Log mother’s wages			− 130.494**			− 139.439**			1.016**			1.086**
			(6.812)			(6.050)			(0.049)			(0.043)
Log other household income				− 203.247**		− 212.759**				1.664**		1.740**
				(10.360)		(9.261)				(0.063)		(0.055)
Log government income					− 8.355	− 10.897					− 0.096	− 0.077
					(9.051)	(7.737)					(0.063)	(0.053)
N (observations)	7352	7352	7352	7352	7352	7352	7352	7352	7352	7352	7352	7352
N (children)	3160	3160	3160	3160	3160	3160	3160	3160	3160	3160	3160	3160
Covariates	N	Y	Y	Y	Y	Y	N	Y	Y	Y	Y	Y

Colombia	Continuous outcome: Poverty gap from national poverty threshold^c^						Continuous outcome: Percentile in the income distribution^d^					
	M1	M2	M3	M4	M5	M6	M1	M2	M3	M4	M5	M6
	Coef (SE)	Coef (SE)	Coef (SE)	Coef (SE)	Coef (SE)	Coef (SE)	Coef (SE)	Coef (SE)	Coef (SE)	Coef (SE)	Coef (SE)	Coef (SE)
Child support receipt	− 55.740**	− 56.937**	− 57.439**	− 55.463**	− 50.484*	− 50.153*	3.826**	3.811**	3.837**	3.694**	3.468**	3.410**
	(21.082)	(21.078)	(21.135)	(20.359)	(20.985)	(20.331)	(0.994)	(0.994)	(0.995)	(0.947)	(0.990)	(0.943)
Log mother’s wages			− 3.062*			− 4.072**			0.179**			0.233**
			(1.351)			(1.299)			(0.061)			(0.057)
Log other household income				− 24.003**		− 23.965**				1.290**		1.290**
				(2.388)		(2.341)				(0.095)		(0.093)
Log government income					− 9.117**	− 8.449**					0.492**	0.457**
					(1.857)	(1.770)					(0.086)	(0.081)
N (observations)	3349	3349	3349	3349	3349	3349	3343	3343	3343	3343	3343	3343
N (children)	1372	1372	1372	1372	1372	1372	1372	1372	1372	1372	1372	1372
Covariates	N	Y	Y	Y	Y	Y	N	Y	Y	Y	Y	Y

*Source*: Authors' calculations based on ELPI (Chile) and ELCA (Colombia)

*Notes*: Sample of children living with a custodial mother in at least two waves. OLS regressions with child and wave fixed effects, standard errors clustered by child. ^a^ Poverty gap from the national poverty line; 0 if above poverty line. ^b^ Calculated using the entirety of survey participants, not just custodial mothers

**Table 10:** Subgroup analyses of child support receipt predicts concurrent poverty status

Chile								
Poverty outcome at t: Measure using national poverty threshold^a^								
	No Step Dad	Step Dad	No Other Adult in HH	Other Adult in HH	More than Secondary	Secondary or Less	Rural	Urban
	Coef (SE)	Coef (SE)	Coef (SE)	Coef (SE)	Coef (SE)	Coef (SE)	Coef (SE)	Coef (SE)
Child support receipt	− 0.085**	− 0.09	− 0.058	− 0.127**	− 0.064*	− 0.099**	− 0.077	− 0.074**
	(0.017)	(0.128)	(0.035)	(0.020)	(0.032)	(0.022)	(0.067)	(0.017)
N (observations)	6842	617	2343	5116	2563	4896	684	6775
N (children	3097	510	1358	2485	1580	2549	370	2964
Poverty outcome at t: Measure using 50% of median income^b^								
	No Step Dad	Step Dad	No Other Adult in HH	Other Adult in HH	More than Secondary	Secondary or Less	Rural	Urban
	Coef (SE)	Coef (SE)	Coef (SE)	Coef (SE)	Coef (SE)	Coef (SE)	Coef (SE)	Coef (SE)
Child support receipt	− 0.099**	− 0.098	− 0.084*	− 0.123**	− 0.071*	− 0.099**	− 0.067	− 0.087**
	(0.017)	(0.121)	(0.037)	(0.019)	(0.029)	(0.023)	(0.074)	(0.016)
N (observations)	6842	617	2343	5116	2563	4896	684	6775
N (children)	3097	510	1358	2485	1580	2549	370	2964

Colombia								
Poverty outcome at t: Measure using national poverty threshold^c^								
	No Step Dad	Step Dad	No Other Adult in HH	Other Adult in HH	More than Secondary	Secondary or Less	Rural	Urban
	Coef (SE)	Coef (SE)	Coef (SE)	Coef (SE)	Coef (SE)	Coef (SE)	Coef (SE)	Coef (SE)
Child support receipt	− 0.057**	− 0.126	− 0.026	− 0.082**	0.067	− 0.083**	− 0.042	− 0.071**
	(0.022)	(0.080)	(0.044)	(0.024)	(0.087)	(0.021)	(0.034)	(0.025)
N (observations)	2851	498	903	2446	499	2850	1302	2047
N (children	1287	328	514	1154	367	1308	566	890
Poverty outcome at t: Measure using 50% of median income^d^								
	No Step Dad	Step Dad	No Other Adult in HH	Other Adult in HH	More than Secondary	Secondary or Less	Rural	Urban
	Coef (SE)	Coef (SE)	Coef (SE)	Coef (SE)	Coef (SE)	Coef (SE)	Coef (SE)	Coef (SE)
Child support receipt	− 0.009	− 0.115	0.032	− 0.018	0.152*	− 0.045 +	− 0.03	− 0.002
	(0.021)	(0.081)	(0.046)	(0.024)	(0.076)	(0.023)	(0.032)	(0.025)
N (observations)	2851	498	903	2446	499	2850	1302	2047
N (children)	1287	328	514	1154	367	1308	566	890

*Source*: Authors’ calculations based on ELPI (Chile) and ELCA (Colombia)

*Notes*: Sample of children living with a custodial mother in at least two waves. Standard errors clustered by child. All models include survey year dummies. Covariates are number of children, dummies for age of youngest child, male child, mother's age at first birth, mother's education dummies, years since bio-father left, and urban. ^a^ In annual US$, the national poverty lines for 2010, 2012, and 2017 respectively are as follows for Chile: 3972, 4380, 4056. ^b^ In annual US$, the 50% of median household income (equivalized) poverty lines for 2010, 2012, and 2017 respectively are as follows for Chile: 3756, 5028, 5682. ^c^ In annual US, the national poverty lines for 2010, 2013, and 2016 respectively are as follows for Rural Colombia: 1224, 1344, 1368. ^d^ In annual US$, the 50% of median household income (equivalized) poverty lines for 2010, 2013, and 2016 respectively are as follows for Colombia: 2460, 3054, 3240

**Table 11:** Child support receipt predicts future childhood poverty status. Sensitivity analysis to sample sizes

Chile	Poverty outcome at t future: Measure using national poverty threshold^a^								Poverty outcome at t future: Measure using 50% of median income^b^							
	M1	M2	M3	M4	M5	M6	Imp. P.^e^	Imp. N.P.^f^	M1	M2	M3	M4	M5	M6	Imp. P.^e^	Imp. N.P.^f^
	Coef (SE)	Coef (SE)	Coef (SE)	Coef (SE)	Coef (SE)	Coef (SE)	Coef (SE)	Coef (SE)	Coef (SE)	Coef (SE)	Coef (SE)	Coef (SE)	Coef (SE)	Coef (SE)	Coef (SE)	Coef (SE)
Child support receipt at t	− 0.065**	− 0.064**	− 0.068**	− 0.067**	− 0.061**	− 0.066**	− 0.036**	− 0.043**	− 0.065**	− 0.064**	− 0.068**	− 0.065**	− 0.060**	− 0.065**	− 0.032*	− 0.039**
	(0.021)	(0.022)	(0.022)	(0.022)	(0.022)	(0.022)	(0.014)	(0.012)	(0.021)	(0.021)	(0.022)	(0.022)	(0.021)	(0.022)	(0.014)	(0.011)
Log mother’s wages at t			− 0.003*			− 0.003*	− 0.002**	− 0.002*			− 0.003*			− 0.003*	− 0.003**	− 0.003**
			(0.001)			(0.001)	(0.001)	(0.001)			(0.001)			(0.001)	(0.001)	(0.001)
Log other household income at t				0.001		0	− 0.002 +	0.001				0.001		0.000	− 0.003*	0.000
				(0.002)		(0.002)	(0.001)	(0.001)				(0.002)		(0.002)	(0.001)	(0.001)
Log government income at t					0.005*	0.005*	0.001	0.003**					0.006**	0.006**	0.002	0.004**
					(0.002)	(0.002)	(0.001)	(0.001)					(0.002)	(0.002)	(0.001)	(0.001)
N (observations)	3417	3417	3417	3417	3417	3417	8203	8203	3417	3417	3417	3417	3417	3417	8203	8203
N (children)	1139	1139	1139	1139	1139	1139	4106	4106	1139	1139	1139	1139	1139	1139	4106	4106
t− 1 Covariates	N	Y	Y	Y	Y	Y	Y	Y	N	Y	Y	Y	Y	Y	Y	Y

Colombia	Poverty outcome at t future: Measure using national poverty threshold^c^								Poverty outcome at t future: Measure using 50% of median income^d^							
	M1	M2	M3	M4	M5	M6	Imp. P.^e^	Imp. N.P.^f^	M1	M2	M3	M4	M5	M6	Imp. P.^e^	Imp. N.P.^f^
	Coef (SE)	Coef (SE)	Coef (SE)	Coef (SE)	Coef (SE)	Coef (SE)	Coef(SE)	Coef(SE)	Coef (SE)	Coef (SE)	Coef (SE)	Coef (SE)	Coef (SE)	Coef (SE)	Coef (SE)	Coef (SE)
Child support receipt at t	− 0.016	− 0.013	− 0.012	− 0.012	− 0.034	− 0.032	− 0.015	− 0.004	− 0.057 +	− 0.03	− 0.034	− 0.031	− 0.052	− 0.056 +	− 0.028	− 0.017
	(0.030)	(0.030)	(0.030)	(0.030)	(0.030)	(0.030)	(0.018)	(0.021)	(0.030)	(0.034)	(0.034)	(0.034)	(0.033)	(0.033)	(0.020)	(0.019)
Log mother’s wages at t			0.001			0.002	− 0.001	0.002			− 0.004*			− 0.004 +	− 0.004**	− 0.002
			(0.002)			(0.002)	(0.001)	(0.001)			(0.002)			(0.002)	(0.001)	(0.001)
Log other household income at t				0.002		0.001	0.000	0.003				− 0.003		− 0.005 +	− 0.004 +	− 0.002
				(0.003)		(0.003)	(0.002)	(0.002)				(0.003)		(0.003)	(0.002)	(0.002)
Log government income at t					0.014**	0.014**	0.006**	0.012**					0.013**	0.013**	0.004**	0.009**
					(0.002)	(0.002)	(0.001)	(0.001)					(0.002)	(0.002)	(0.001)	(0.001)
N (observations)	1815	1815	1815	1815	1815	1815	3669	3669	1815	1815	1815	1815	1815	1815	3669	3669
N (children)	605	605	605	605	605	605	1902	1902	605	605	605	605	605	605	1902	1902
t-1 Covariates	N	Y	Y	Y	Y	Y	Y	Y	N	Y	Y	Y	Y	Y	Y	Y

*Source*: Authors' calculations based on ELPI (Chile) and ELCA (Colombia)

*Notes*: Sample of children living with a custodial mother in all waves for M1-M6. Sample of children living with a custodial mother in at least one wave Imp. P. & Imp. N.P. Logit models with standard errors clustered by child, margins at means reported. All models control for poverty status in base year, a dummy for the timeframe used, and months between surveys. Covariates from t are number of children, dummies for age of youngest child, male child, mother's age at first birth, mother's education dummies, years since bio-father left, and urban. t is wave 1 except for observations using wave 2 and wave 3, in which case t is wave 2. ^a^ In annual US$, the national poverty lines for 2010, 2012, and 2017 respectively are as follows for Chile: 3972, 4380, 4056. ^b^ In annual US$, the 50% of median household income (equivalized) poverty lines for 2010, 2012, and 2017 respectively are as follows for Chile: 3756, 5028, 5682. ^c^ In annual US, the national poverty lines for 2010, 2013, and 2016 respectively are as follows for Rural Colombia: 1224, 1344, 1368. ^d^ In annual US$, the 50% of median household income (equivalized) poverty lines for 2010, 2013, and 2016 respectively are as follows for Colombia: 2460, 3054, 3240. ^e^ Imp. P. = Imputed Poor. Assigns all attrited outcomes as poor at t future. Same specification as M6 but with a larger sample including attritors. ^f^ Imp. N.P. = Imputed Not Poor. Assigns all attrited outcomes as not poor at t future. Same specification as M6 but with a larger sample including attritors

**Table 12:** Child support receipt predicts future continuous outcomes

Chile	Continuous outcome at t future: Poverty gap from national poverty threshold^a^						Continuous outcome at t future: Percentile in the income distribution^b^					
	M1	M2	M3	M4	M5	M6	M1	M2	M3	M4	M5	M6
	Coef (SE)	Coef (SE)	Coef (SE)	Coef (SE)	Coef (SE)	Coef (SE)	Coef (SE)	Coef (SE)	Coef (SE)	Coef (SE)	Coef (SE)	Coef (SE)
Child support receipt at t	− 636.992**	− 633.776**	− 684.255**	− 706.360**	− 628.548**	− 721.570**	3.773**	3.668**	3.772**	4.016**	3.481**	3.830**
	(114.273)	(114.391)	(114.608)	(116.259)	(114.019)	(115.777)	(0.834)	(0.827)	(0.830)	(0.838)	(0.825)	(0.836)
Log mother’s wages at t			− 32.627**			− 27.463**			0.067			0.033
			(7.170)			(7.326)			(0.050)			(0.052)
Log other household income at t				41.471**		28.090*				− 0.199*		− 0.167*
				(10.837)		(11.035)				(0.078)		(0.081)
Log government income at t					9.906	7.316					− 0.354**	− 0.343**
					(11.818)	(11.822)					(0.085)	(0.085)
N (observations)	5285	5285	5285	5285	5285	5285	5285	5285	5285	5285	5285	5285
N (children)	3099	3099	3099	3099	3099	3099	3099	3099	3099	3099	3099	3099
t-1 Covariates	N	Y	Y	Y	Y	Y	N	Y	Y	Y	Y	Y

Colombia	Continuous outcome at t future: Poverty gap from national poverty threshold^c^						Continuous outcome at t future: Percentile in the income distribution^d^					
	M1	M2	M3	M4	M5	M6	M1	M2	M3	M4	M5	M6
	Coef (SE)	Coef (SE)	Coef (SE)	Coef (SE)	Coef (SE)	Coef (SE)	Coef (SE)	Coef (SE)	Coef (SE)	Coef (SE)	Coef (SE)	Coef (SE)
Child support receipt at t	38.473	27.851	27.771	28.635	14.353	14.394	0.237	0.556	0.541	0.459	1.348	1.258
	(24.866)	(24.230)	(24.221)	(24.249)	(23.259)	(23.282)	(1.082)	(1.080)	(1.076)	(1.072)	(1.047)	(1.041)
Log mother’s wages at t			1.96			2.620 +			0.282**			0.239**
			(1.369)			(1.354)			(0.062)			(0.060)
Log other household income at t				2.054		0.743				− 0.256**		− 0.121
				(2.126)		(2.098)				(0.096)		(0.094)
Log government income at t					10.820**	10.928**					− 0.643**	− 0.617**
					(1.593)	(1.600)					(0.072)	(0.072)
N (observations)	2576	2576	2576	2576	2576	2576	2572	2572	2572	2572	2572	2572
N (children)	1370	1370	1370	1370	1370	1370	1370	1370	1370	1370	1370	1370
t-1 Covariates	N	Y	Y	Y	Y	Y	N	Y	Y	Y	Y	Y

*Source*: Authors’ calculations based on ELPI (Chile) and ELCA (Colombia)

*Notes*: Sample with children living with a custodial mother in at least two waves. Logit models with standard errors clustered by child, margins at means reported. All models control for poverty status in base year, a dummy for the timeframe used, and months between surveys. Covariates from t are number of children, dummies for age of youngest child, male child, mother's age at first birth, mother's education dummies, years since bio-father left, and urban. t is wave 1 except for observations using wave 2 and wave 3, in which case t is wave 2. ^a^ In annual US$, the national poverty lines for 2010, 2012, and 2017 respectively are as follows for Chile: 3972, 4380, 4056. ^b^ In annual US$, the 50% of median household income (equivalized) poverty lines for 2010, 2012, and 2017 respectively are as follows for Chile: 3756, 5028, 5682. ^c^ In annual US, the national poverty lines for 2010, 2013, and 2016 respectively are as follows for Rural Colombia: 1224, 1344, 1368. ^d^ In annual US$, the 50% of median household income (equivalized) poverty lines for 2010, 2013, and 2016 respectively are as follows for Colombia: 2460, 3054, 3240

**Table 13:** Child support receipt predicts future childhood poverty status. Subgroup analyses

Chile										
Poverty outcome at t future: Measure using national poverty threshold^a^										
	No Step Dad	Step Dad	No Other Adult in HH	Other Adult in HH	More than Secondary	Secondary or Less	Rural	Urban	Not Poor	Poor
	Coef (SE)	Coef (SE)	Coef (SE)	Coef (SE)	Coef (SE)	Coef (SE)	Coef (SE)	Coef (SE)	Coef (SE)	Coef (SE)
Child support receipt at t	− 0.072**	0.004	− 0.155**	− 0.056**	− 0.090**	− 0.057**	− 0.08	− 0.071**	− 0.057**	− 0.082**
	(0.017)	(0.081)	(0.040)	(0.018)	(0.025)	(0.020)	(0.056)	(0.017)	(0.020)	(0.023)
N (observations)	5205	233	1325	4113	1688	3750	515	4923	2708	2730
N (children)	3056	180	862	2408	1164	2325	338	2919	1779	1783
Poverty outcome at t future: Measure using 50% of median income^b^										
	No Step Dad	Step Dad	No Other Adult in HH	Other Adult in HH	More than Secondary	Secondary or Less	Rural	Urban	Not Poor	Poor
	Coef (SE)	Coef (SE)	Coef (SE)	Coef (SE)	Coef (SE)	Coef (SE)	Coef (SE)	Coef (SE)	Coef (SE)	Coef (SE)
Child support receipt at t	− 0.067**	0.043	− 0.157**	− 0.051**	− 0.083**	− 0.053**	− 0.123*	− 0.059**	− 0.064**	− 0.062*
	(0.017)	(0.081)	(0.039)	(0.018)	(0.025)	(0.020)	(0.057)	(0.017)	(0.018)	(0.029)
N (observations)	5205	233	1325	4113	1688	3750	515	4923	3749	1689
N (children)	3056	180	862	2408	1164	2325	338	2919	2322	1183

Colombia										
Poverty outcome at t future: Measure using national poverty threshold^c^										
	No Step Dad	Step Dad	No Other Adult in HH	Other Adult in HH	More than Secondary	Secondary or Less	Rural	Urban	Not Poor	Poor
	Coef (SE)	Coef (SE)	Coef (SE)	Coef (SE)	Coef (SE)	Coef (SE)	Coef (SE)	Coef (SE)	Coef (SE)	Coef (SE)
Child support receipt at t	− 0.017	− 0.158 +	− 0.079	− 0.01	− 0.072	− 0.012	− 0.05	− 0.007	0.022	− 0.038
	(0.026)	(0.095)	(0.057)	(0.028)	(0.054)	(0.025)	(0.037)	(0.034)	(0.040)	(0.024)
N (observations)	2323	259	595	1987	326	2256	1068	1514	668	1914
N (children	1267	182	375	1107	260	1219	552	840	460	1058
Poverty outcome at t future: Measure using 50% of median income^d^										
	No Step Dad	Step Dad	No Other Adult in HH	Other Adult in HH	More than Secondary	Secondary or Less	Rural	Urban	Not Poor	Poor
	Coef (SE)	Coef (SE)	Coef (SE)	Coef (SE)	Coef (SE)	Coef (SE)	Coef (SE)	Coef (SE)	Coef (SE)	Coef (SE)
Child support receipt at t	− 0.048 +	0.021	− 0.048	− 0.044	− 0.006	− 0.04	− 0.06	− 0.024	− 0.038	− 0.035
	(0.027)	(0.107)	(0.062)	(0.029)	(0.030)	(0.028)	(0.038)	(0.026)	(0.026)	(0.033)
N (observations)	2323	259	595	1987	326	2256	1068	1514	1194	1388
N (children)	1267	182	375	1107	260	1219	552	840	760	784

*Source*: Authors’ calculations based on ELPI (Chile) and ELCA (Colombia)

*Notes*: Sample of children living with a custodial mother in at least two waves. Logit models with standard errors clustered by child, margins at means reported. All models control for poverty status in base year, a dummy for the timeframe used, and months between surveys. Covariates from t are log(mother’s wages), log(other household income), and log(government income), number of children, dummies for age of youngest child, male child, mother's age at first birth, mother's education dummies, years since bio-father left, and urban. t is wave 1 except for observations using wave 2 and wave 3, in which case t is wave 2. ^a^ In annual US$, the national poverty lines for 2010, 2012, and 2017 respectively are as follows for Chile: 3972, 4380, 4056. ^b^ In annual US$, the 50% of median household income (equivalized) poverty lines for 2010, 2012, and 2017 respectively are as follows for Chile: 3756, 5028, 5682. ^c^ In annual US, the national poverty lines for 2010, 2013, and 2016 respectively are as follows for Rural Colombia: 1224, 1344, 1368. ^d^ In annual US$, the 50% of median household income (equivalized) poverty lines for 2010, 2013, and 2016 respectively are as follows for Colombia: 2460, 3054, 3240

**Table 14:** Child support receipt predicts future childhood poverty status using different timeframes for future childhood poverty

Panel A: Short term	Chile 2010–2012		Colombia 2010–2013	
	Poverty outcome		Poverty outcome	
	National poverty line^a^	50% Median Income^b^	National Poverty Line^c^	50% Median Income^d^
	Coef./SE	Coef./SE	Coef./SE	Coef./SE
2010 Child support receipt	− 0.053	− 0.064*	− 0.044	− 0.089 +
	(0.033)	(0.029)	(0.043)	(0.051)
2010 Log mother's wages	0	− 0.002	0	− 0.005 +
	(0.002)	(0.002)	(0.003)	(0.003)
2010 Log other household income	− 0.001	− 0.001	− 0.002	− 0.005
	(0.003)	(0.003)	(0.003)	(0.004)
2010 Log government income	0.030**	0.024**	0.016**	0.015**
	(0.007)	(0.006)	(0.003)	(0.003)
N(children)	1139	1139	605	605

Panel B: Long term	Chile 2010–2017		Colombia 2010–2016	
	Poverty outcome		Poverty outcome	
	National Poverty Line^a^	50% Median Income^b^	National Poverty Line^c^	50% Median Income^d^
	Coef (SE)	Coef (SE)	Coef (SE)	Coef (SE)
2010 Child support receipt	− 0.052 +	− 0.043	− 0.035	− 0.084 +
	(0.031)	(0.031)	(0.047)	(0.049)
2010 Log mother's wages	− 0.005*	− 0.004 +	− 0.008*	− 0.011**
	(0.002)	(0.002)	(0.003)	(0.003)
2010 Log other household income	− 0.001	− 0.001	− 0.001	0.002
	(0.003)	(0.003)	(0.004)	(0.004)
2010 Log government income	0.017*	0.017*	0.012**	0.012**
	(0.007)	(0.007)	(0.003)	(0.003)
N(children)	1139	1139	605	605

*Source*: Authors’ calculations based on ELPI (Chile) and ELCA (Colombia)

*Notes*: Sample of children living with a custodial mother in all waves. Logit models with standard errors clustered by child, margins at means reported. All models control for poverty status in base year, a dummy for the timeframe used, and months between surveys. Covariates from t are number of children, dummies for age of youngest child, male child, mother's age at first birth, mother's education dummies, years since bio-father left, and urban. t is wave 1 except for observations using wave 2 and wave 3, in which case t is wave 2. ^a^ In annual US$, the national poverty lines for 2010, 2012, and 2017 respectively are as follows for Chile: 3972, 4380, 4056. ^b^ In annual US$, the 50% of median household income (equivalized) poverty lines for 2010, 2012, and 2017 respectively are as follows for Chile: 3756, 5028, 5682. ^c^ In annual US, the national poverty lines for 2010, 2013, and 2016 respectively are as follows for Rural Colombia: 1224, 1344, 1368. ^d^ In annual US$, the 50% of median household income (equivalized) poverty lines for 2010, 2013, and 2016 respectively are as follows for Colombia: 2460, 3054, 3240

## Footnotes

1 In Chile, one child was surveyed per household/mother.

2 Prior research with the Chilean data from the 2010 survey round also finds around 50% of monetary poverty (Narea, 2014). Estimates with the Colombian data from 2010 show a household poverty rate of 39.8% in urban areas and 49% in rural areas. Although our rates are somewhat higher, a number of studies have found that custodial-mother families are disproportionately poor as compared to two-parent families and the general population (Cuesta & Guarin, 2024; Salinas, 2011). We see similar trends to those shown in the national poverty measures: over the time period under investigation, they halved.

3 52% in 2010, 20% in 2013, and 26% in 2016.

4 Currently, we use the government transfer amount provided in the survey.

5 The coefficients we report estimate the magnitude of how a change from non-receipt to receipt reduces the probability of child poverty. With our cross-sectional data, we use the margins command after logit to generate these results. Because marginal effects can be problematic when applying fixed effects in the panel setting (Kemp & Santos Silva, 2016), we use OLS even though we have a binary outcome. OLS specifications coefficients have the same interpretation as marginal effects and have been shown to be comparable to logit estimations with sufficient variation of the outcome variable (i.e. not < 10% or > 90%) (Hellevik, 2009).

6 It is important to note that national poverty was falling in both countries over this period.

## Code Availability

The analyses were done using Stata 14. If accepted, do files for replication can be included as supplementary material or posted online on the author’s website, as preferred by the editors.
